# HPV16 integration regulates ferroptosis resistance via the c-Myc/miR-142-5p/HOXA5/SLC7A11 axis during cervical carcinogenesis

**DOI:** 10.1186/s13578-024-01309-2

**Published:** 2024-10-17

**Authors:** Xiao-Jing Chen, Chu-Hong Guo, Yang Yang, Zi-Ci Wang, Yun-Yi Liang, Yong-Qi Cai, Xiao-Feng Cui, Liang-Sheng Fan, Wei Wang

**Affiliations:** 1 Department of Obstetrics and Gynecology, The First Affiliated Hospital of Guangzhou Medical University, 151 Yanjiang Road, Yuexiu District, Guangzhou, 510120 People’s Republic of China; 2 Department of Gynecology, Guangdong Provincial People’s Hospital (Guangdong Academy of Medical Sciences), Southern Medical University, Guangzhou, 510080 People’s Republic of China; 3 Department of Obstetrics and Gynecology, Affiliated Qingyuan Hospital, Guangzhou Medical University, Qingyuan, 511699 People’s Republic of China; 4 Department of Gynecology, Shanghai Key Laboratory of Maternal Fetal Medicine, Shanghai Institute of Maternal-Fetal Medicine and Gynecologic Oncology, Shanghai First Maternity and Infant Hospital, School of Medicine, Tongji University, Shanghai, 201204 People’s Republic of China

**Keywords:** HPV16 integration, Ferroptosis, Cervical carcinogenesis, C-Myc, SLC7A11

## Abstract

**Background:**

Ferroptosis, a newly identified form of regulated cell death triggered by small molecules or specific conditions, plays a significant role in virus-associated carcinogenesis. However, whether tumours arising after high-risk HPV integration are associated with ferroptosis is unexplored and remains enigmatic.

**Methods:**

High-risk HPV16 integration was analysed by high­throughput viral integration detection (HIVID). Ferroptosis was induced by erastin, and the levels of ferroptosis were assessed through the measurement of lipid-reactive oxygen species (ROS), malondialdehyde (MDA), intracellular Fe2^+^ level and transmission electron microscopy (TEM). Additionally, clinical cervical specimens and an in vivo xenograft model were utilized for the study.

**Results:**

Expression of HPV16 integration hot spot c-Myc negatively correlates with ferroptosis during the progression of cervical squamous cell carcinoma (CSCC). Further investigation revealed that the upregulated oncogene miR-142-5p in HPV16-integrated CSCC cells served as a critical downstream effector of c-Myc in its target network. Inhibiting miR-142-5p significantly decreased the ferroptosis-suppressing effect mediated by c-Myc. Through a combination of computational and experimental approaches, HOXA5 was identified as a key downstream target gene of miR-142-5p. Overexpression of miR-142-5p suppressed HOXA5 expression, leading to decreased accumulation of intracellular Fe2^+^ and lipid peroxides (ROS and MDA). HOXA5 increased the sensitivity of CSCC cells to erastin-induced ferroptosis via transcriptional downregulation of SLC7A11, a negative regulator of ferroptosis. Importantly, c-Myc knockdown increased the anti-tumour activity of erastin by promoting ferroptosis both in vitro and in vivo.

**Conclusions:**

Collectively, these data indicate that HPV16 integration hot spot c-Myc plays a novel and indispensable role in ferroptosis resistance by regulating the miR-142-5p/HOXA5/SLC7A11 signalling axis and suggest a potential therapeutic approach for HPV16 integration-related CSCC.

**Supplementary Information:**

The online version contains supplementary material available at 10.1186/s13578-024-01309-2.

## Introduction

Cervical carcinogenesis is primarily caused by human papillomavirus (HPV) [1], and the integration of viral DNA into the host cell genome is frequently observed in both high-grade lesions and invasive cancers [2]. Our previous research indicated that HPV16, the most prevalent high-risk HPV associated with cervical squamous cell carcinoma (CSCC), has a tendency to integrate into transcriptionally active regions and fragile sites [3]. Numerous genes involved in cancer development are positioned at or in close proximity to the sites where HPV16 integrates, suggesting their potential involvement in cervical carcinogenesis [4]. Importantly, we and others have shown that the c-Myc chromosomal locus is the most frequently affected site of HPV16 integration [3, 5]. The activation of c-Myc due to the insertion of HPV16 DNA sequences could be a critical genetic event in carcinogenesis [6]. Thus, elucidating the regulatory mechanisms of c-Myc is expected to yield insight into HPV16-induced cervical carcinogenesis.

The dysregulation of cell proliferation and death dynamics during viral infection plays a critical role in tumorigenesis. Cancer cells utilize intricate mechanisms to survive viral assaults, such as impeding cell death pathways like necroptosis, autophagy, and apoptosis [7]. Ferroptosis, a newly identified form of regulated cell demise, is initiated by the accumulation of lipid peroxides in an iron-dependent manner [8]. The compound erastin can trigger ferroptosis by inhibiting the cystine/glutamate antiporter system Xc −, leading to heightened lipid peroxide levels [9]. However, negative regulators of ferroptosis within cells may hamper the therapeutic application of ferroptosis inducers [10]. Solute carrier family 7 member 11 (SLC7A11), a critical element of system Xc −, mitigates lipid peroxidation by transporting cystine into the cytosol, fostering glutathione (GSH) production, and thwarting ferroptosis [11]. Therefore, knockdown of SLC7A11 is proposed to make cancer cells vulnerable to ferroptosis. Emerging data suggest that elevated SLC7A11 expression in precancerous lesions and tumor tissues correlates with adverse outcomes and therapy resistance [12]. The upregulation of SLC7A11 is triggered by physiological factors like inflammation and hypoxia, affecting intracellular GSH levels [13]. However, whether HPV16 integration regulates SLC7A11 expression to suppress ferroptotic cell death and increase the longevity of HPV16-infected cells in the local environment remains inadequately elucidated.

miRNAs are small noncoding RNAs that pair with the 3′-UTRs of target mRNAs, leading to posttranscriptional modulation of target gene expression [14]. Anomalies in miRNA production are strongly connected to viral infections and tumorigenesis [15]. Our recent analysis using a miRNA array in HPV16-integrated CSCC cells revealed an increase in miR-142-5p levels [16]. Remarkably, miR-142-5p participates in a range of biological processes, such as differentiation, apoptosis, and autophagy, and has been linked to multiple diseases, including cancer [17]. However, the exact functions of miR-142-5p in modulating ferroptosis and its association with HPV16 integration-related carcinogenesis remain unexplored.

In this study, we aimed to investigate whether tumours arising after HPV16 integration are associated with ferroptosis. And explore the underlying mechanisms for HPV16 integration in regulating ferroptosis resistance during cervical carcinogenesis, which may provide potential targets for the prevention and treatment of CSCC.

## Materials and methods

### Cell lines and culture

The SiHa and CaSki cell lines, both containing HPV16 integration, were procured from the American Type Culture Collection (ATCC). The HaCaT cell line, an HPV16 non-integrated immortalized human cervical keratinocyte cell line, was obtained from the China Center for Type Culture Collection (CCTCC) and maintained according to standard protocols. The S12 cell line, an HPV16-integrated immortalized human cervical keratinocyte cell line, was graciously provided by *Prof.* Hu Zheng (Precision Medicine Institute, the First Affiliated Hospital, Sun Yat-sen University, Guangzhou, China) [3], with permission from the original owner, *Prof.* Margaret Stanley (Department of Pathology, University of Cambridge, Cambridge, United Kingdom) [18]. S12 cells were cultured in a 1:3 mixture of DMEM and Ham’s F-12 medium supplemented with 5% FBS, 8.4 ng/ml cholera toxin, 5 μg/ml insulin, 0.5 μg/ml hydrocortisone, 24.3 μg/ml adenine, and 10 ng/ml epidermal growth factor. Further details regarding cell line authentication are available in the supplementary materials.

### Clinical specimens

Clinical specimens were collected from the Department of Gynecological Oncology at the First Affiliated Hospital of Guangzhou Medical University, comprising a total of 100 cervical samples. The specimens included 20 normal cervix, 20 cervical intraepithelial neoplasia (CIN) I, 30 CIN II-III, and 30 CSCC tissues. Lesion samples were sourced from cervical biopsies (8 cases), loop electrosurgical excision procedures (22 cases), cone biopsies (25 cases), and radical hysterectomies (25 cases). Additionally, 20 normal cervical tissue specimens were obtained from patients undergoing hysterectomy for non-cancerous conditions, with no history of CIN or abnormal Pap smears. Pregnant women, individuals with acute pelvic infections, and those who had undergone radiotherapy/chemotherapy were excluded from the study. The study involving human tissue samples obtained ethical approval from the Ethics Committee of the First Affiliated Hospital of Guangzhou Medical University (approval number: 2022-58, granted on 14 April 2022) and adhered to the principles outlined in the Declaration of Helsinki. Informed consent was waived due to the use of residual specimens and routine medical records. Each sample was assessed by two experienced pathologists.

### High­throughput viral integration detection (HIVID)

HPV16 integration was analyzed using the HIVID method, as presented in Table 1. HIVID is a next­generation sequencing and computational method developed by our collaborative research group [19]. While primarily used for HBV integration, HIVID has also been successfully applied to detect integration of other viruses, including HPV, as demonstrated in our previous study [3]. The detailed experimental procedure has been previously documented in our previous study. Briefly, the methodology encompassed the design of sequence-capture probes targeting 17 distinct HPV genome sequences (6, 11, 16, 18, 31, 33, 35, 39, 45, 52, 56, 58, 59, 66, 68, 69, and 82) supplied by MyGenostics. The HIVID pipeline was deployed for breakpoint identification, with RNA-seq employed for precise breakpoint localization. Subsequent PCR amplification was facilitated using the GeneAmp PCR System 9700 thermal cycler, with Sanger sequencing conducted on the Applied Biosystems 3730 × DNA analyzer (Life Technologies Inc.). The identified integrated breakpoints were annotated via ANNOVAR [20].

**Table 1:** HPV16 integration, c-Myc and SLC7A11 expression in different cervical tissues

Type	n	HPV16 integration		c-Myc		SLC7A11	
		Positive	%	Positive	%	Positive	%
Nomal	20	0	0	0	0	0	0
CIN I	20	2	10.0	5	25.0	4	20.0
CIN II-III	30	8	26.7	19	63.3	17	56.7
CSCC	30	13	43.3	26	86.7	23	76.7

*CIN* cervical intraepithelial neoplasia, *CSCC* cervical squamous cell carcinoma

### RNA extraction and RT-qPCR

RNA extraction from cells was performed using the miRNeasy Mini Kit (Qiagen), following the manufacturer’s guidelines. For miRNA, reverse transcription adopted the Mir-X™ miRNA First-Strand Synthesis Kit (TaKaRa), while for mRNA, the PrimeScript™ RT Master Mix (TaKaRa) was employed. RT-qPCR was performed as previously described [21]. Primer sets for miR-142-5p and U6 were sourced from RiboBio Inc. [16]. Expression levels of both miRNAs and mRNAs were standardized against U6 and GAPDH, respectively. Refer to Supplemental Table 1 for the primer sequences.

### Western blot analysis

Western blot analysis was conducted following established protocols [21]. Primary antibodies included anti-SLC7A11 (#ab37185, Abcam), anti-HOXA5 (#ab140636, Abcam), and anti-GAPDH (#60004-1-Ig, Proteintech). Secondary antibodies applied were horseradish peroxidase-conjugated anti-rabbit (#ab6721, Abcam) and anti-mouse (#ab6789, Abcam) immunoglobulin-G. In order to avoid interference caused by simultaneous exposure of different protein molecules with different expression levels, we performed complete cleavage and exposure analysis of the bands based on different molecular weights.

### Dual luciferase assays

To explore the interaction between c-Myc and the miR-142-5p promoter, the coding region of c-Myc wild sequence (WT) or its mutated biding sequence (MT), and the 2-kb region upstream of the miR-142-5p transcription start site were PCR amplified and inserted into pcDNA3.1 (+) and PGL3 vectors, respectively. For assessing miR-142-5p targeting of HOXA5, the potential miR-142-5p complementary site in the 3′-UTR of HOXA5 or its mutant sequence was cloned into the pmiR-RB-Report vector (RiboBio Inc.). Subsequently, pmiR-RB-Report-HOXA5-3′UTR-WT or pmiR-RB-Report- HOXA5-3′UTR-MT were co-transfected into S12/SiHa/CaSki cells with miR-142-5p mimics. To investigate HOXA5 targeting of SLC7A11, SLC7A11 promoter plasmids with firefly luciferase reporters were co-transfected with an internal control pRL-TK containing a full-length Renilla luciferase gene (GeneChem Inc.) into S12/SiHa/CaSki cells overexpressing HOXA5, following the manufacturer’s guidelines. After 48 h post-transfection, the cells were analyzed using the Dual-Luciferase Reporter Assay System (Promega). Firefly luciferase activity was normalized to Renilla luciferase activity for each well. All experiments were performed in triplicate and repeated thrice. The cloning sequences can be found in Supplemental Table 2.

### Chromatin immunoprecipitation (ChIP) assay

Cells were fixed using 1% formaldehyde, quenched with glycine, and then subjected to a ChIP assay using an Enzymatic ChIP Kit (#9003, CST) following the provided protocols [16]. Immunoprecipitation was carried out with anti-HOXA5 (#sc-365784X, SantaCruz) and control IgG (#2729, CST) antibodies. The enrichment of HOXA5-binding sites (HBS) within the SLC7A11 promoter region was assessed through qPCR. The results were presented as the relative enrichment normalized to control IgG. Detailed primer sequences utilized for ChIP-PCR are listed in Supplemental Table 1.

### miRNA target prediction

The prediction and analysis of miRNA targets were performed utilizing the algorithms TargetScan (http://www.targetscan.org/), PicTar (http://pictar.bio.nyu.edu/), and miRWalk (http://zmf.umm.uni-heidelberg.de/apps/zmf/mirwalk2/) [16].

### In situ hybridization (ISH) and immunohistochemical (IHC) staining

ISH was conducted on cervical tissues utilizing an ISH kit and miR-142-5p synthetic oligonucleotide probes (Boster Biological Technology Co., Ltd) according to the manufacturer’s instructions [16]. Briefly, slides were processed with 3% H_2_O_2_, digested with pepsin, and fixed with 1% PFA in DEPC. Subsequently, the slides were pre-hybridized with hybridization buffer at 42 °C using miR-142-5p or U6 probes, followed by incubation with streptavidin–biotin complex and horseradish peroxidase polymer. For IHC staining [21], sections were treated with 3% H_2_O_2_ to block endogenous peroxidase activity, and incubated with primary antibodies overnight at 4 °C. A horseradish peroxidase-conjugated anti-rabbit secondary antibody was applied for 1 h at 26 °C. The expression of c-Myc (#ab32072, Abcam), SLC7A11 (#ab37185, Abcam), and HOXA5 (#ab140636, Abcam) was visualized using DAB and counterstained with hematoxylin. ISH and IHC tissue sections underwent independent evaluation and scoring by two experienced pathologists. The H-score algorithm was applied as previously detailed [21]. The median H-score values were used to categorize groups with low and high expression of c-Myc or SLC7A11.

### Transient transfection with oligonucleotides and plasmids

Mimic/inhibitor chemically modified oligonucleotides were utilized to imitate or suppress endogenous miRNAs. The miR-142-5p mimic/inhibitor and corresponding negative controls were designed and synthesized by RiboBio Inc. Transfection with the miR-142-5p mimic or inhibitor was employed to regulate the levels of miR-142-5p. The HOXA5 coding sequence (without the 3′-UTR) was inserted into the pCDNA3.1 (+) vector (RiboBio Inc.). An empty vector served as the control. Cells were assessed for RNA extraction, western blot analysis, and in vitro assays 48 h after transfection.

### Lipid reactive oxygen species (ROS) measurements

Cellular lipid ROS levels were assessed using a ROS Assay Kit (#S0033S, Beyotime) as per the manufacturer’s protocol [22]. Cells were plated in a 6-well plate and treated with serum-free medium containing 10 μmol/L 2′,7′-dichlorodihydrofluorescein diacetate (DCFH-DA) in the absence of light for 20 min at 37 °C with periodic agitation. Subsequently, cells were collected, rinsed with serum-free medium, and re-suspended in serum-free medium. Measurement of fluorescence intensity at an excitation wavelength of 488 nm and an emission wavelength of 525 nm allowed for the evaluation of intracellular ROS levels. Serum-free medium was utilized to prevent potential interference from endogenous esterase activity in serum [23].

### Malondialdehyde (MDA) assay

The MDA content in cell lysates was quantified using a Lipid Peroxidation MDA Assay Kit (#S0131S, Beyotime) following the manufacturer’s protocol [24]. Cells were harvested and lysed with cell RIPA Lysis Buffer (#KGP702, KeyGEN). The resulting supernatant, obtained post-centrifugation at 10,000–12,000 g for 10 min, was utilized for the analysis. TBA was added to the samples to form MDA-TBA adducts, and the relative MDA concentration was determined colorimetrically at an absorbance of 535 nm.

### Transmission electron microscopy (TEM)

To prepare for electron microscopy, cells were first fixed with 2% glutaraldehyde for 5 min [25]. Subsequently, postfixation was performed using osmium tetroxide, followed by dehydration with ethanol, propylene oxide treatment, and embedding in Spurr’s epoxy resin. Thin sections of 90 nm were stained with uranyl acetate and lead citrate before being examined under an H-7500 transmission electron microscope (Hitachi) at a magnification of 40,000 ×. Ferroptotic cells typically display an increase in mitochondrial membrane density along with a reduction or loss of cristae and outer mitochondrial membranes [26].

### Iron assay

The levels of intracellular ferrous iron (Fe2 +) were assessed using the iron assay kit (ab83366, Abcom) as per the instructions provided by the manufacturer [24]. Samples were gathered, rinsed with chilled PBS, and homogenized in 5 × volumes of iron assay buffer on ice. The resulting supernatant was then treated with iron reducer and iron probe, and each sample was subsequently incubated. The measurement was taken using a colorimetric microplate reader, reading the output at an optical density of 593 nm.

### Colony formation assay

For the colony formation assay [27], S12 and SiHa cells were plated in 60 mm dishes. Upon reaching 70–80% confluence, the cells were either transduced with lentivirus carrying c-Myc overexpression or exposed to the c-Myc antagomir (IZCZ-3). Following trypsinization and cell counting, the cells were re-seeded at appropriate dilutions to allow for colony formation. After an incubation period of 10–14 days, the formed colonies were fixed, stained with crystal violet, washed, and air-dried. The plating efficiency was calculated for each cell line, and the survival rate was determined based on the number of colonies formed post-treatment. Each experiment was conducted in triplicate.

### Establishment of mCherry overexpression SiHa cells

To establish SiHa cells overexpressing mCherry, lentiviral vector containing the mCherry sequence (lenti-mCherry) was obtained from GeneChem Inc. SiHa cells were transfected with lenti-mCherry as per the manufacturer’s protocol [25]. Purinomycin (2 μg/ml) was added 48 h after virus transfection for selection. The culture medium with purinomycin was refreshed every 2–3 days over a 4-week period. The transfection efficiency was subsequently assessed using a fluorescence microscope.

### In vivo xenograft model

Six-week-old immunodeficient Female nude mice were obtained from Guangdong Animal Center (Guangzhou, China) and randomly divided into four groups (n = 3 mice/group): (a) NC group; (b) IZCZ-3 group; (c) erastin group; and (d) IZCZ-3 + erastin group. To establish subcutaneous tumors in mice, SiHa-mCherry cells (5 × 10^6^ cells/mouse) were subcutaneously injected into the right posterior flanks of nude mice without matrigel. Tumor size was recorded every 4 days, and tumor volume was calculated using the formula: volume (mm^3^) = 1/2 (length × width^2^). When the tumor volume reached approximately 50 mm^3^, mice were intraperitoneally treated with 10 mg/kg IZCZ-3 (MCE, #HY-111411) and/or 10 mg/kg erastin (MCE, #HY-15763) daily for 20 days. IZCZ-3 and erastin were dissolved in a solution of 5% dimethyl sulfoxide (DMSO) and corn oil. Animal experiments were approved by the Committee Review of Animal Experiments in Guangzhou Medical University (approved number: 2019-081, on 26 February 2019) and were performed in accordance with the Basel Declaration.

### Statistical analysis

Statistical analysis was performed using SPSS software (version 20.0). The results are presented as the mean ± SEM values and were analyzed using t-tests or one-way ANOVA. Frequency tables were analyzed using the chi-squared test, while the Pearson correlation coefficient was employed to evaluate the significance of links between categorical variables. Statistical significance was defined as a P-value below 0.05.

## Results

### Expression of the HPV16 integration hot spot c-Myc negatively correlates with ferroptosis during the progression of CSCC

To explore the association between HPV16 integration and ferroptosis during cervical carcinogenesis, we conducted HIVID for HPV integration detection and performed IHC staining for c-Myc and SLC7A11 (a known negative regulator of ferroptosis) expression in 20 normal cervix, 20 CIN I, 30 CIN II-III, and 30 CSCC tissues. The HPV16 integration rates in the respective histological groups were 0%, 10.0%, 26.7%, and 43.3%, showing a progressive increase with higher histological grades. Similarly, the rates of c-Myc and SLC7A11 expression also demonstrated a gradual rise from normal cervical epithelium to CSCC (Table 1).

**Fig. 1:**
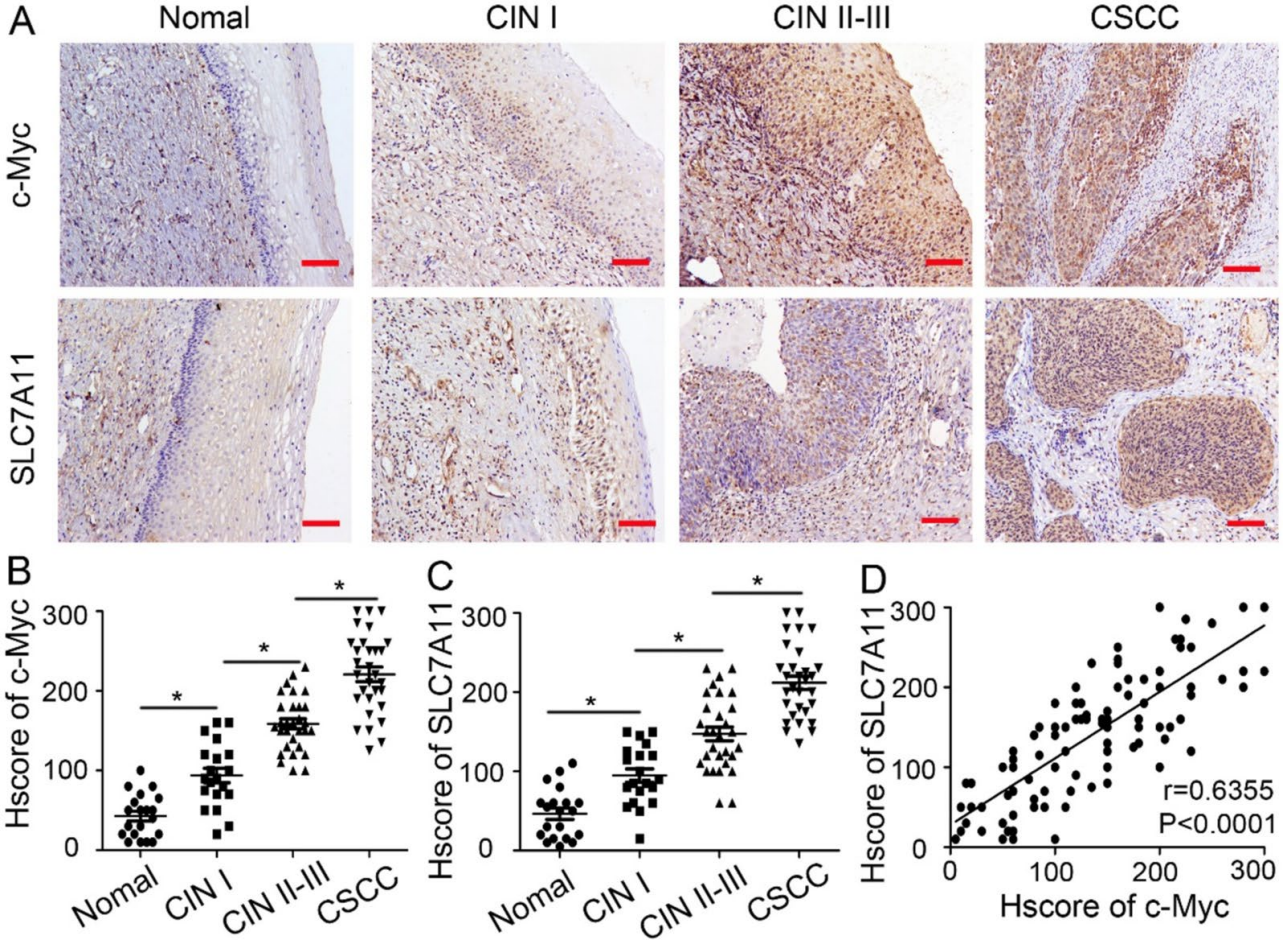
The correlation between c-Myc and ferroptosis during CSCC progression. **A** Representative IHC staining of c-Myc and SLC7A11 in normal cervix, CIN I, CIN II-III, and CSCC tissues (n = 20, n = 20, n = 30, n = 30 respectively). The images were captured at 200 × magnification, with a scale bar of 50 μm. **B, C** Statistical analysis of c-Myc and SLC7A11 expression in different tissue samples. *P < 0.05. **D** Pearson correlation analysis was used to evaluate the clinical relevance of c-Myc and SLC7A11 (r = 0.6360, *P* < 0.0001)

As depicted in Fig. 1A, c-Myc was observed in the nucleus of cervical epithelial cells, while SLC7A11 exhibited membrane staining. Immunoreactivity of c-Myc and SLC7A11 significantly intensified from normal cervical epithelium to dysplasia and CSCC stages (200 × magnification). The expression levels of c-Myc and SLC7A11 were significantly higher in CSCC compared to both CIN grades (both *P* < 0.05), and were higher in both CIN grades compared to normal cervical epithelium (both* P* < 0.05). Additionally, a significant difference was noted between early- and late-stage CIN tissues (both *P* < 0.05) (Fig. 1B, C). Clinically, we evaluated the correlation between c-Myc and SLC7A11 expression using Pearson correlation analysis, revealing a significant positive correlation (Fig. 1D; r = 0.6355, *P* < 0.0001). These results suggest that expression of the HPV16 integration hot spot c-Myc negatively correlates with ferroptosis during the progression of CSCC.

### c-Myc regulates miR-142-5p to suppress ferroptosis

**Fig. 2:**
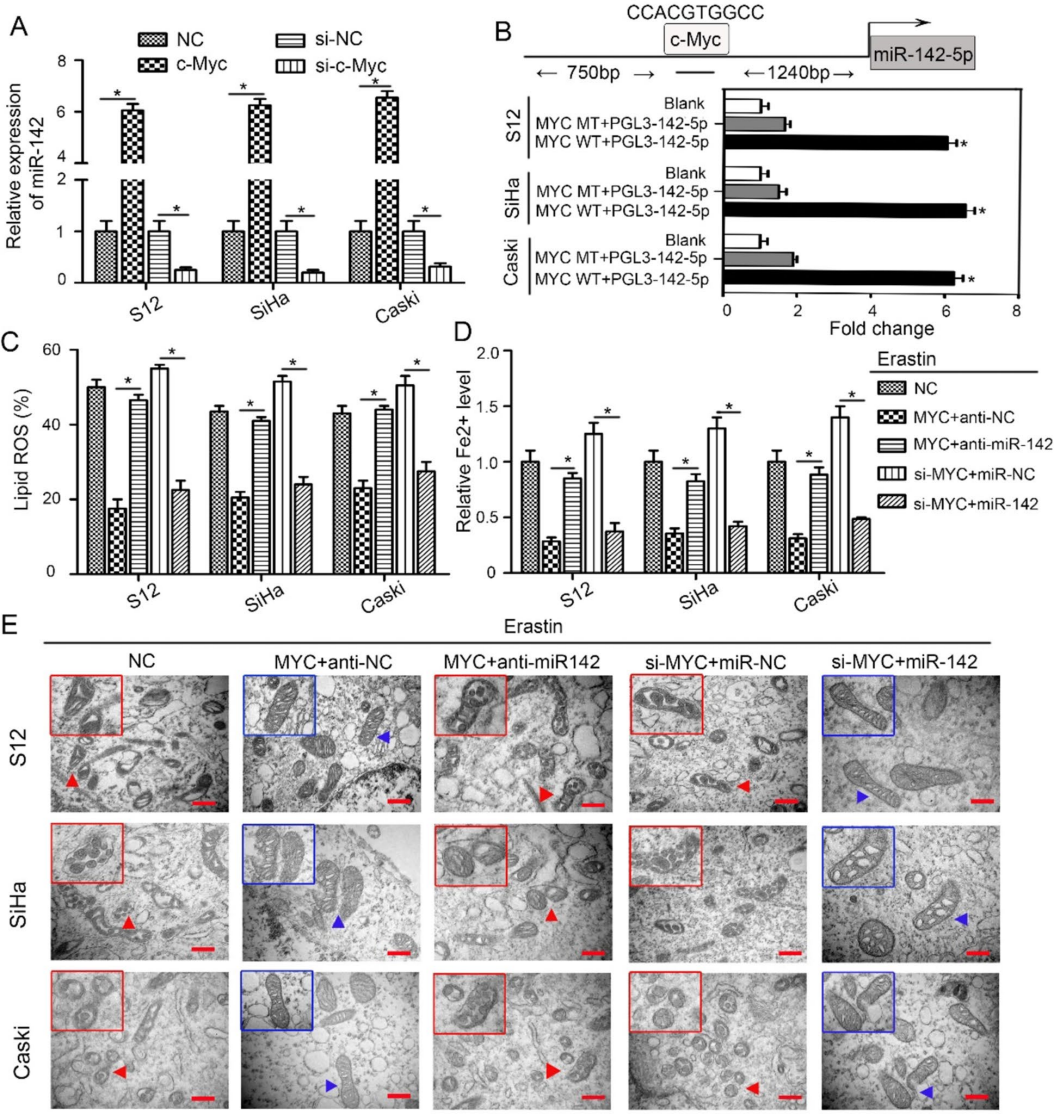
c-Myc regulates miR-142-5p to suppress ferroptosis. **A** RT-qPCR analysis of miR-142-5p expression in S12, SiHa, and CaSki cells with c-Myc overexpression or knockdown. *P < 0.05. **B** Schematic representation of the miR-142-5p upstream promoter containing the c-Myc binding site. Luciferase activity of the PGL3-142-5p construct after transfection of the c-Myc WT (wild sequence) and MT (the mutated c-Myc binding site) plasmid in S12, SiHa, and CaSki cells. *P < 0.05. **C**–**E** S12, SiHa, and CaSki cells were transfected with c-Myc overexpression vector with or without miR-142-5p inhibitor, or with c-Myc siRNA with or without miR-142-5p mimic. Cells were then treated with 10 μM erastin for 24 h. Lipid ROS levels (**C**) and intracellular Fe^2+^ levels (**D**) were quantified using commercial kits. *P < 0.05. **E** Representative TEM images displaying mitochondrial morphology of S12, SiHa and CaSki cells in response to erastin stimulation were captured, with blue arrows indicating normal mitochondria and red arrows pointing to ferroptotic mitochondria characterized by decreased volume, increased membrane density, and disrupted cristae structure. The images were captured at 40,000 × magnification

Our prior study revealed that miR-142-5p is highly expressed in HPV16-integrated CSCC cells [16]. Considering that c-Myc functions as the transcriptionally active region for HPV16 integration [3], it is likely to exert significant control over the expression of miR-142-5p. To investigate this, we employed HPV16-integrated cervical epithelial cells (S12, SiHa, and CaSki) with c-Myc overexpression and knockdown (Supplemental Fig. 1). The results indicated that miR-142-5p expression increased in c-Myc-overexpressing cells and decreased in c-Myc-silenced cells, suggesting that c-Myc controls miR-142-5p expression in HPV16-integrated cervical epithelial cells (Fig. 2A). To understand the mechanism underlying the regulation of miR-142-5p by c-Myc, we cloned the miR-142-5p promoter into the pGL3-Basic vector and conducted a dual-luciferase reporter assay to examine the interaction between c-Myc and miR-142-5p. Transient expression of c-Myc WT effectively stimulated the transcription of miR-142-5p than c-Myc MT (the mutated c-Myc binding site) in S12, SiHa and CaSki cells (Fig. 2B, P < 0.05). This implies that c-Myc directly targets the miR-142-5p promoter in HPV16-integrated cervical epithelial cells.

To further validate the role of the c-Myc/miR-142-5p axis in ferroptosis, we conducted experiments using both HPV16-integrated cells (S12, SiHa, and CaSki) as well as HPV16 non-integrated HaCat cells. These cells were transfected with a c-Myc overexpression vector, with or without the miR-142-5p inhibitor, or with c-Myc siRNA, with or without the miR-142-5p mimic. Iron-dependent and lipid peroxides accumulation are indispensable process for ferroptosis [28]. The intracellular level of ferrous iron (Fe2 +) was measured using an iron assay kit from Abcam. As lipid ROS and MDA are key end products of lipid peroxidation [29], we also assessed whether the c-Myc/miR-142-5p axis regulates lipid ROS generation and MDA production following erastin treatment. The results, as shown in Fig. 2C, D and Supplemental Fig. 2, 3, demonstrate that the increases in ROS, MDA levels, and Fe2 + accumulation induced by erastin were notably reduced by c-Myc overexpression. Nevertheless, this inhibitory effect was reversed when cells were co-transfected with the miR-142-5p inhibitor. c-Myc silencing also increased intracellular Fe^2+^ and lipid peroxides accumulation, but these effects were abrogated in cells co-transfected with the miR-142-5p mimic. To further confirm the ultrastructural changes associated with ferroptosis, transmission electron microscopy (TEM) was performed. Morphological alterations characteristic of ferroptotic cells, such as distinct mitochondrial changes including reduced volume, heightened membrane density, and loss of mitochondrial cristae, were observed in erastin-treated S12, SiHa, and CaSki cells (marked with red arrows in Fig. 2E). However, c-Myc overexpression significantly reduced erastin-induced ferroptotic cell death (indicated by blue arrows in Fig. 2E), and co-transfection with the miR-142-5p inhibitor increased the mitochondrial membrane density even in the presence of c-Myc. Conversely, knockdown of c-Myc using RNA interference increased mitochondrial membrane density, but this ferroptotic effect was nullified by co-transfection with the miR-142-5p mimic. These data confirm that the HPV16 integration hot spot c-Myc regulates miR-142-5p to suppress ferroptosis.

### The 3′-UTR of HOXA5 is a direct target of miR-142-5p

**Fig. 3:**
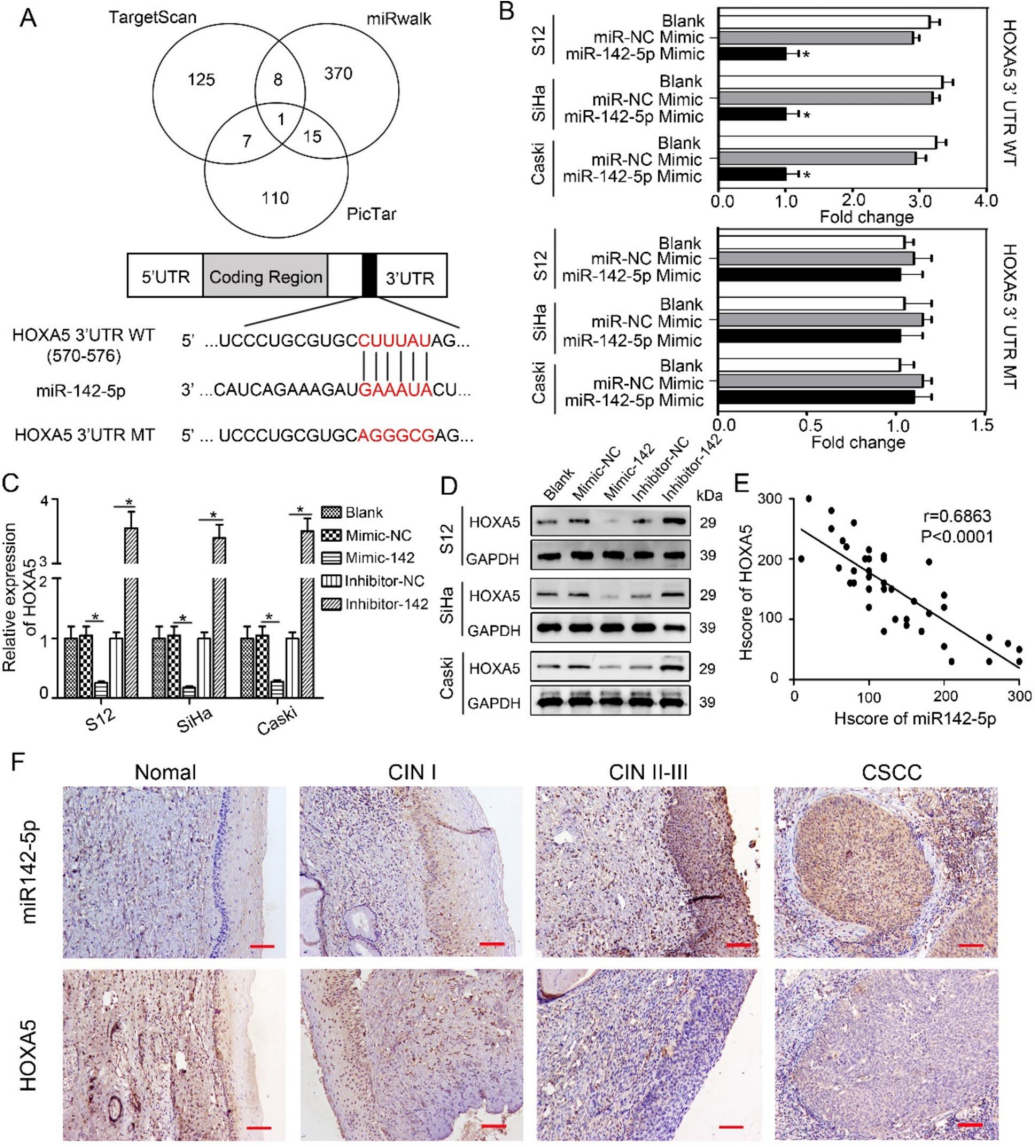
The 3′-UTR of HOXA5 is a direct target of miR-142-5p. **A** The overlapping targets of miR-142-5p predicted by three distinct miRNA target prediction algorithms were presented, highlighting the wild-type and mutated binding sites between miR-142-5p and HOXA5. **B** Following transfection of the miR-142-5p mimic or miR-NC mimic, the luciferase activity of the wild-type 3′UTR-HOXA5-luc and mutant 3′UTR-HOXA5-luc constructs was assessed in S12, SiHa, and CaSki cells. **C, D** The expression levels of HOXA5 in these cells following transfection with the miR-142-5p mimic, miR-NC mimic, miR-142-5p inhibitor, and miR-NC inhibitor were investigated using both RT-qPCR and western blotting. **E** Pearson correlation analysis was conducted to assess the clinical significance of miR-142-5p and HOXA5 (r = − 0.7213, *P* < 0.0001). **F** Representative micrographs displayed the immunohistochemical staining patterns of miR-142-5p and HOXA5 in normal cervix, CIN I, CIN II-III, and CSCC tissues, observed at a magnification of 200 × (scale bar, 50 μm)

To identify specific targets of miR-142-5p, we utilized publicly available bioinformatics tools, including TargetScan, PicTar, and miRWalk, to screen oxidative stress genes with potential miR-142-5p binding sites in their 3′-UTRs. All three algorithms predicted potential miR-142-5p binding sites in the 3′-UTR of HOXA5 (Fig. 3A). To validate this prediction, we inserted the 3′-UTR sequence of HOXA5 into a luciferase reporter plasmid, introducing a mismatch mutation in the seed sequence to generate mutant reporter vectors. Upregulation of miR-142-5p significantly decreased the luciferase activity of the wild-type HOXA5 3′-UTR in S12, SiHa and CaSki cells (Fig. 3B, *P < 0.05). However, mutant HOXA5 3′-UTR blocked this inhibitory effect of miR-142-5p (Fig. 3B, P > 0.05). These findings indicate that miR-142-5p directly targets the 3′-UTR of HOXA5.

Furthermore, we investigated the influence of miR-142-5p on the mRNA and protein levels of HOXA5 using RT-qPCR and western blotting, respectively. Upregulation of miR-142-5p led to a notable reduction in both the mRNA and protein levels of HOXA5. Conversely, suppression of miR-142-5p by an inhibitor increased the mRNA and protein expression levels of HOXA5 (Fig. 3C, D), suggesting that miR-142-5p regulates HOXA5 expression in S12, SiHa and CaSki cells. Additionally, we analyzed the expression levels of miR-142-5p and HOXA5 in cervical specimens through ISH and IHC staining, respectively (Fig. 3E, F). Elevated immunoreactivity of miR-142-5p was detected in more advanced cervical lesions (at 200 × magnification). Conversely, HOXA5 staining progressively diminished with increasing histological grade. Pearson correlation analysis revealed a significant negative correlation between miR-142-5p and HOXA5 expression (r = 0.6863, *P* < 0.0001). Collectively, these findings indicate that deficiency of HOXA5 is associated with CSCC progression and that miR-142-5p is responsible for the abnormal expression of HOXA5 in HPV16-integrated cervical epithelial cells.

### The miR-142-5p/HOXA5 axis regulates ferroptosis in CSCC cells by targeting SLC7A11

**Fig. 4:**
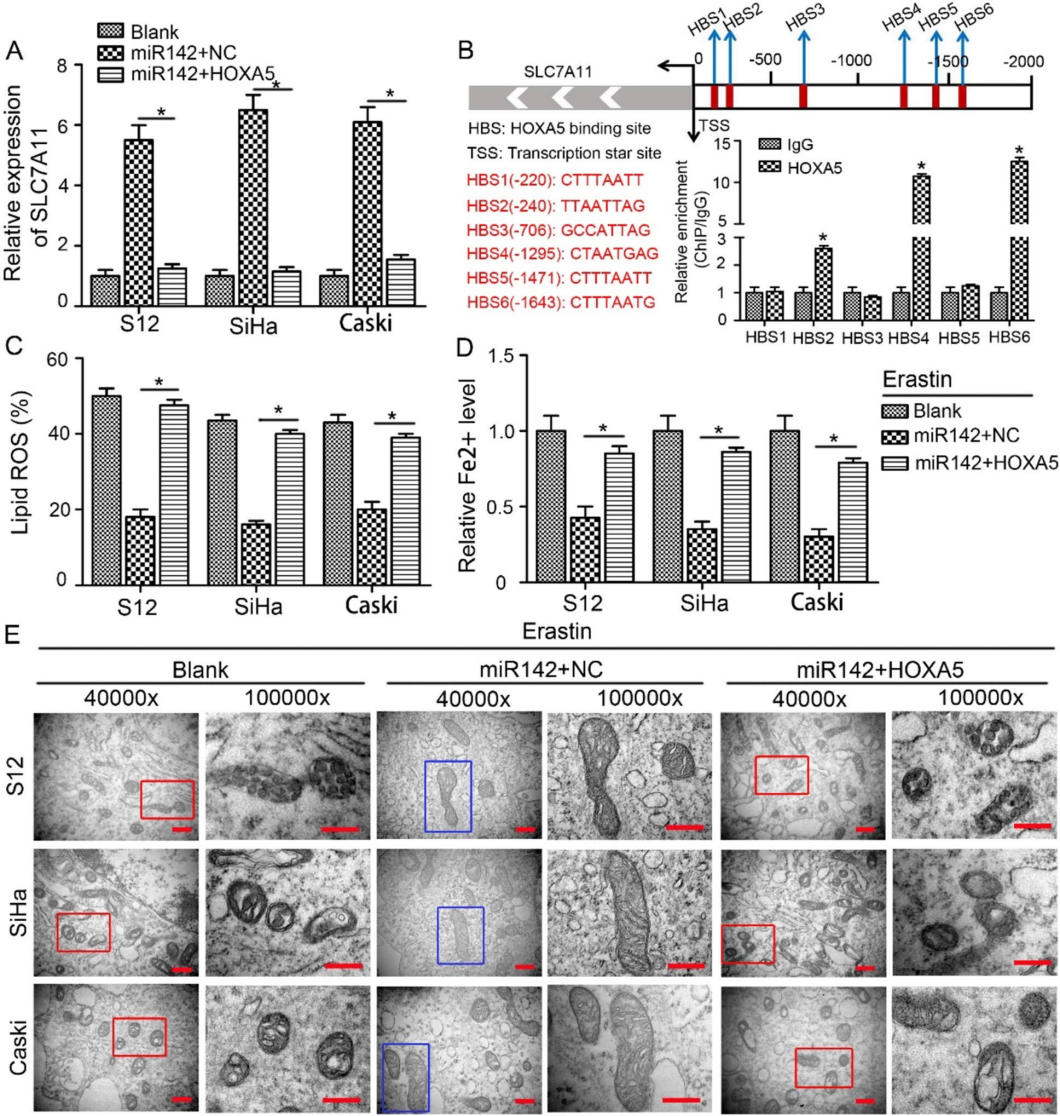
The miR-142-5p/HOXA5 axis regulates ferroptosis in CSCC cells by targeting SLC7A11. **A** RT-qPCR analysis of SLC7A11 expression in S12, SiHa, and CaSki cells transfected with the miR-142-5p mimic, with or without the HOXA5 overexpression vector. *P < 0.05. **B** A schematic depiction illustrated the predicted binding sites of HOXA5 on the SLC7A11 promoter. ChIP-PCR assays were executed to ascertain the enrichment of HOXA5 on the designated HOXA5 binding sites (HBS) within the SLC7A11 promoter zone in comparison to IgG. **C–E** HPV16-integrated cells (S12, SiHa, and CaSki) were transfected with the miR-142-5p mimic, with or without the HOXA5 overexpression vector, followed by treatment with 10 μM erastin for 24 h. Lipid ROS levels (**C**) and intracellular Fe^2+^ levels (**D**) were measured with the corresponding commercial kits. *P < 0.05. **E** Representative TEM images demonstrating mitochondrial morphology in S12, SiHa, and CaSki cells post-erastin stimulation. Normal mitochondria were outlined in blue boxes, while ferroptotic mitochondria, characterized by reduced volume, increased membrane density, and disappeared cristae, were highlighted in red boxes. The images were captured at magnifications of 40,000 × and 100,000 ×, respectively (scale bar, 500 nm)

HOXA5 is a crucial transcription factor with diverse functions [30]. We further performed rescue experiments to analyse whether HOXA5 modulates ferroptosis by targeting SLC7A11. The coding sequence of HOXA5 was inserted into an expression vector lacking the miR-142-5p binding site, making it impervious to miRNA-induced downregulation. Transfection with the miR-142-5p mimic significantly elevated the mRNA and protein levels of SLC7A11 in S12, SiHa, and CaSki cells, which were subsequently suppressed by co-transfection with the HOXA5 overexpression vector (Fig. 4A and Supplemental Fig. 4A). These findings demonstrated that exogenous supplementation of HOXA5 was sufficient to counteract the effect of miR-142-5p and decrease the expression level of SLC7A11. In order to elucidate the underlying molecular mechanisms of HOXA5-mediated SLC7A11 expression, an analysis of the JASPAR database was performed [31], which predicted the presence of six binding sites for HOXA5 (HBS) in the promoter region of SLC7A11. Luciferase reporter assays showed a decrease in SLC7A11 promoter-driven luciferase activity in cells overexpressing HOXA5 (Supplemental Fig. 4B, *P < 0.05). Additionally, ChIP-PCR assays were conducted to investigate the occupancy of the SLC7A11 promoter region (from − 220 to − 1643 bp upstream of the exon) by HOXA5. Strong interaction between HOXA5 and three binding sites (HBS2, HBS4, and HBS6) was observed (Fig. 4B, *P < 0.05), indicating that HOXA5 directly inhibits the transcription of SLC7A11.

As HOXA5 mediated the expression of SLC7A11, we further explored the role of miR-142-5p/HOXA5 axis in ferroptosis. Subsequent transfection with the miR-142-5p mimic effectively attenuated erastin-induced intracellular accumulation of Fe2 + and lipid peroxides (ROS and MDA) in S12, SiHa, and CaSki cells (Fig. 4C, D and Supplemental Fig. 5). However, this inhibitory effect was reversed upon co-transfection with the HOXA5 overexpression vector. TEM analysis was utilized to confirm the ultrastructural alterations associated with ferroptosis, aligning with the previous observations (Fig. 4E, blue boxes indicating normal mitochondria and red boxes indicating ferroptotic mitochondria). Collectively, these results indicate that the miR-142-5p/HOXA5 axis regulates ferroptosis in HPV16-integrated cervical epithelial cells through the targeting SLC7A11.

### Combination treatment with c-Myc inhibitor and ferroptosis inducer represses CSCC growth

**Fig. 5:**
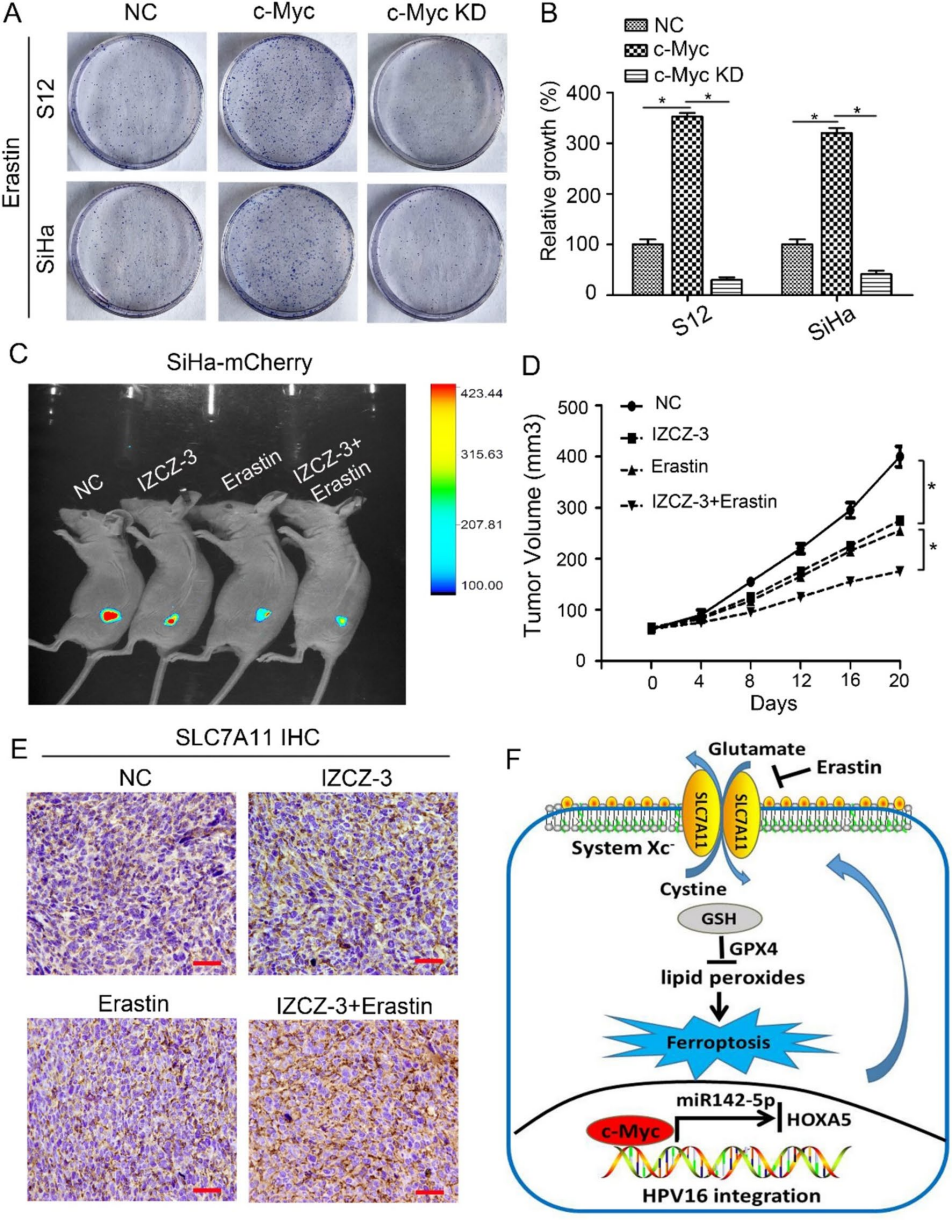
Combination treatment with c-Myc inhibitor and ferroptosis inducer represses CSCC growth. **A**, **B** Colony formation assay of the indicated S12 and SiHa cell lines. Overexpression and silencing of c-Myc were achieved through lentiviral transduction and antagomir (IZCZ-3) techniques, respectively. Subsequently, cells were exposed to erastin (10 µM) for 24 h and then cultured without erastin for 14 days. All dishes for each cell line were fixed, stained, and imaged simultaneously. *P < 0.05. **C**, **D** Suppression of c-Myc enhances erastin-induced ferroptosis in vivo. Nude mice were subcutaneously injected with SiHa-mCherry cells (5 × 10^6^ cells/mouse). Treatment with 10 mg/kg IZCZ-3 and/or 10 mg/kg erastin was administered intraperitoneally daily for 20 days when the tumor volume reached around 50 mm^3^. Tumor volume measurements were taken every 4 days. The data are presented as the mean ± SD values (n = 3 mice/group). *P < 0.05. **E** Representative IHC images showing the expression level of SLC7A11 in mouse tumor sections of each group. The images were captured at a magnification of 200 × (scale bar, 50 μm). **F** A schematic illustration depicting c-Myc-mediated ferroptosis resistance in HPV16-integrated cervical epithelial cells. The HPV16 integration hot spot c-Myc suppresses ferroptosis by regulating the miR-142-5p/HOXA5/SLC7A11 signalling axis

The observed effects of c-Myc on ferroptosis suggest that the expression level of c-Myc modulates the antitumour activity of erastin. Thus, we overexpressed or silenced c-Myc expression in S12 and SiHa cells, followed by treatment with erastin and assessment of cell viability through colony formation analysis. Notably, the enforced elevation of c-Myc resulted in a marked reduction in ferroptotic cell death induced by erastin. Conversely, the inhibition of endogenous c-Myc through the use of antagomir (IZCZ-3) significantly intensified erastin-induced ferroptotic cell death (Fig. 5A, B). The expression levels of SLC7A11 in the indicated S12 and SiHa cells were measured by western blot and RT-qPCR, confirming that c-Myc indirectly modulates SLC7A11 levels, predominantly through miR-142-5p (Supplemental Fig. 6).

We next investigated whether knockdown of c-Myc enhances erastin-induced ferroptotic cell death in vivo. Because S12 cells cannot form tumours, the animal model of CSCC was established with SiHa cells. SiHa-mCherry cells were implanted into the subcutaneous space of nude mice. When the tumour volume was approximately 50 mm^3^, the mice were randomly divided into four groups and treated intraperitoneally with IZCZ-3 and/or erastin daily for 20 days. Repression of c-Myc or induction of ferroptosis reduced the size of the formed tumours compared with those in the control group. Moreover, the tumours in mice treated with the combination of IZCZ-3 and erastin were markedly smaller than those in the other groups (Fig. 5C, D). And we performed histopathology analysis to further detect the expression level of SLC7A11 in mouse tumor sections of each group (Fig. 5E). The expression level of SLC7A11 was significantly higher in the combined administration group (IZCZ + Erastin) than in the single administration group (either IZCZ or Erastin) and control group. These results suggest that the combination treatment with c-Myc antagomir and ferroptosis inducer can effectively induce ferroptosis and repress CSCC growth in vivo. In summary, HPV16 integration hot spot c-Myc plays a novel and indispensable role in ferroptosis resistance by regulating the miR-142-5p/HOXA5/SLC7A11 signalling axis (Fig. 5F), suggesting a potential therapeutic approach for HPV16 integration-related CSCC.

## Discussion

Ferroptosis, a novel form of non-apoptotic cell death, involves iron-dependent lipid peroxide accumulation and leads to lethal cellular damage [28]. Targeting ferroptosis-related pathways has shown promise in developing effective anti-cancer treatments [32]. Consequently, significant efforts have been made to uncover the regulatory mechanisms of ferroptosis and its involvement in tumorigenesis [33]. Our previous research focused on investigating the processes of cervical cancer development driven by HPV16 integration and identifying specific integration sites in the human genome [3]. However, there is currently no research available on the viral integration-mediated regulation of ferroptosis. In this study, we demonstrate that HPV16 integration induces ferroptosis resistance through the c-Myc/miR-142-5p/HOXA5/SLC7A11 signalling axis and thus promotes cervical carcinogenesis. These data link viral integration to ferroptosis during carcinogenesis and are expected to provide potential targets for the prevention and treatment of CSCC.

In an extensive study of HPV-associated tumors, it was observed that viral integration sites were predominantly located within transcriptionally active regions [34]. Furthermore, fragile sites have been identified as facilitators of HPV DNA integration, indicating a non-random and deliberate process [35]. Although a wide range of integration sites have been documented, the c-Myc chromosomal locus stands out as a common insertion site for hr-HPV [36]. HPV18, frequently linked to adenocarcinoma, tends to integrate at the c-Myc locus [37]. Notably, recent observations by our research team and others have highlighted HPV16 DNA integration at this locus as well [3, 38]. The regulation of c-Myc expression is intricate and involves genetic amplifications, insertions, and translocations [39]. Our study revealed that the insertion of HPV16 DNA sequences triggers the activation of c-Myc, marking a significant genetic event in cervical carcinogenesis. Other factors, including TIP30 [40], E2F1 [41], and KPNA2 [42], can also regulate c-Myc expression through transcriptional mechanisms. The activation of c-Myc driven by HPV16 integration in epithelial cells may influence the differentiation of transformed keratinocytes in concert with microenvironmental interactions, partially contributing to the high incidence of HPV16-induced CSCC. This cellular process not only supports viral maintenance but also represents an irreversible step in cell transformation. Through its diverse targets, c-Myc serves as a crucial regulator of essential cellular functions such as proliferation, apoptosis, differentiation, and DNA metabolism [43]. However, the role of c-Myc in ferroptosis processes remains unclear. Here, we demonstrated that c-Myc expression is inversely correlated with the occurrence of ferroptosis during CSCC progression. Silencing c-Myc enhanced the susceptibility of CSCC cells to erastin-induced ferroptosis, significantly impairing their colony-forming ability in vitro and tumor development in a xenograft mouse model. These data presented here concerning the most frequently identified HPV16 DNA integration site suggest that the alteration of the c-Myc gene due to HPV16 DNA integration contributes to the suppression of ferroptosis, thereby promoting the progression of CSCC. Thus, combination therapies targeting c-Myc and ferroptosis inducers may offer a comprehensive therapeutic approach for CSCC.

MicroRNAs are pivotal players in cancer development, functioning either as oncogenes or tumor suppressor genes [44]^.^ Our prior research demonstrated the upregulation of miR-142-5p as a potent oncogene in HPV16-integrated CSCC cells [16]. The current investigation highlights the involvement of the HPV16 integration hotspot, c-Myc, in stimulating the transcriptional activity and expression of miR-142-5p by directly interacting with its promoter region. Notably, miR-142-5p induced by c-Myc serves as a crucial mediator in regulating ferroptosis. Inhibition of miR-142-5p could depress the c-Myc-induced ferroptosis resistance, as indicated by decreased intracellular Fe2^+^ and lipid peroxides in c-Myc overexpression cells treated with miR-142-5p inhibitors. Conversely, restoration of miR-142-5p promoted the biological functions of CSCC cells, which recapitulated the effects of c-Myc knockdown. In addition to its impact on ferroptosis, it is recognized that miR-142-5p participates in the regulation of apoptosis [45] and autophagy [46]. Unlike autophagy and apoptosis, miR-142-5p-mediated ferroptosis is characterized by an iron and ROS-dependent process leading to cytological changes, including mitochondrial cristae depletion, disrupted outer mitochondrial membrane, and condensed mitochondrial membrane [8, 26]. These cellular alterations result from compromised membrane permeability due to extensive lipid peroxidation and elevated oxidative stress [47]. Further exploration into the pivotal role of miR-142-5p in the interplay between apoptosis, autophagy, and ferroptosis could yield significant clinical implications, given the close association between evading cell death and tumorigenesis as well as drug resistance.

Numerous genes have been identified as direct targets of miR-142-5p [17]. For example, our prior study revealed that miR-142-5p specifically targets ARID2, resulting in the remodeling of peri-tumoral lymphatic vessels and metastatic dissemination [16]. In this study, we focused on HOXA5 as a potentially significant downstream target of miR-142-5p. Our luciferase reporter assay results have validated HOXA5 as a newly identified direct target of miR-142-5p. HOXA5 is known for its involvement in transcriptional regulation, cellular differentiation, and carcinogenesis. Previous studies have identified HOXA5 as a tumor suppressor in various cancers [48, 49]. However, the precise molecular mechanisms through which HOXA5 influences CSCC development remain elusive. Our data illustrate that miR-142-5p overexpression leads to a notable decrease in HOXA5 expression. Additionally, overexpression of HOXA5 counteracted the promotive effect of miR-142-5p on ferroptosis resistance, confirming that miR-142-5p modulates ferroptosis through its inhibitory effects on HOXA5. These findings deepen our comprehension of miR-142-5p’s role in tumorigenesis and propose a potential therapeutic avenue.

SLC7A11, the active component of system Xc −, serves as a crucial oncogenic protein involved in defending against oxidative stress [50] and ferroptosis [11], affecting malignant behavior, immune response, and treatment sensitivity [11]. Emerging evidence suggests that there is a close correlation between ferroptosis and oxidative stress [51]. Oxidative stress induces ferroptosis by generating a large amount of ROS promoting the accumulation of lipid peroxides. In addition, the process of ferroptosis is accompanied by oxidative stress, which promotes each other and jointly leads to cell damage and death [52]. Importantly, SLC7A11 has emerged as a central hub linking ferroptosis to oxidative stress. In this study, we found that HPV16 integration induced ferroptosis resistance in cervical epithelial cells. And this ferroptosis resistance effect was abrogated in cells treated with the SLC7A11 inhibitor erastin. Therefore, we identified SLC7A11 as a key molecule for inhibiting ferroptosis in the microenvironment after HPV16 integration infection. Targeting SLC7A11 has shown promise in several studies. For example, genetic ablation or pharmacological inhibition of SLC7A11 strongly induces ferroptosis in various cancer cell types, whereas SLC7A11 overexpression in cancer cells promotes glutathione biosynthesis, confers ferroptosis resistance, and is associated with unfavorable patient outcomes [12]. Therefore, exploring the regulatory mechanisms of SLC7A11 has been a very important focus. The expression and function of SLC7A11 are intricately controlled through a variety of mechanisms, such as transcriptional regulation by transcription factors and epigenetic regulators, as well as post-transcriptional mechanisms [11]. Among these, one of the important mechanisms involves the competitive binding of positive and negative transcription factors to an antioxidant response element (ARE) in the SLC7A11 promoter [53]. Current evidence indicates that viral miRNAs may target negative transcriptional regulators of ARE-associated genes [54]. Therefore, we investigated whether SLC7A11 could be a downstream effector of the miR-142-5p/HOXA5 axis. Our results demonstrated that enforced miR-142-5p expression reduced HOXA5 levels, leading to ferroptosis resistance through increased SLC7A11 expression. Our data confirmed that HOXA5 acts as an upstream transcription factor of SLC7A11, providing insights for developing rational combination therapies involving SLC7A11 inhibitors for therapeutic purposes.

## Conclusions

In conclusion, our data provide novel and significant evidence for the association of HPV16 integration with ferroptosis resistance during cervical carcinogenesis. The HPV16 integration hot spot c-Myc regulates miR-142-5p to suppress ferroptosis. Elevated levels of miR-142-5p in HPV16-integrated cervical epithelial cells directly suppress HOXA5, resulting in increased transcription of SLC7A11, which aids in resistance to ferroptosis and the progression of cervical squamous cell carcinoma (CSCC). The newly identified c-Myc/miR-142-5p/HOXA5/SLC7A11 signalling axis in HPV16-integrated cervical epithelial cells appears to be a critical molecular mechanism underlying CSCC progression and could serve as a novel diagnostic and therapeutic target in CSCC.

## Supplementary Information

Additional file 1

## Data Availability

All data generated or analyzed during this study are included in this published article.

## Abbreviations

CSCC: Cervical squamous cell carcinoma
ROS: Reactive oxygen species
MDA: Malondialdehyde
HPV: Human papillomavirus
SLC7A11: Solute carrier family 7 member 11
GSH: Glutathione
ATCC: American type culture collection
CCTCC: China center for type culture collection
CIN: Cervical intraepithelial neoplasia
HIVID: High­throughput viral integration detection
ChIP: Chromatin immunoprecipitation
HBS: HOXA5-binding sites
ROS: Lipid reactive oxygen species
MDA: Malondialdehyde
DCFH-DA: 2′, 7′-Dichlorodihydrofluorescein diacetate
DCF: Deesterify dichlorofluorescein
TBA: Thiobarbituric acid
Fe^2+^: Intracellular ferrous iron
ISH: In situ hybridization
IHC: Immunohistochemical

## References

[1] Wang X, Huang X, Zhang Y. Involvement of human papillomaviruses in cervical cancer. Front Microbiol. 2018;9:2896. 30546351 10.3389/fmicb.2018.02896 PMC6279876

[2] Cancer Genome Atlas Research Network, et al. Integrated genomic and molecular characterization of cervical cancer. Nature. 2017; 543: 378–384. 10.1038/nature21386 PMC5354998 28112728

[3] Hu Z, et al. Genome-wide profiling of HPV integration in cervical cancer identifies clustered genomic hot spots and a potential microhomology-mediated integration mechanism. Nat Genet. 2015;47:158–63. 25581428 10.1038/ng.3178

[4] Ojesina AI, et al. Landscape of genomic alterations in cervical carcinomas. Nature. 2014;506:371–5. 24390348 10.1038/nature12881 PMC4161954

[5] Peter M, Rosty C, Couturier J, Radvanyi F, Teshima H, Sastre-Garau X. MYC activation associated with the integration of HPV DNA at the MYC locus in genital tumors. Oncogene. 2006;25:5985–93. 16682952 10.1038/sj.onc.1209625

[6] Gabay M, Li Y, Felsher DW. MYC activation is a hallmark of cancer initiation and maintenance. Cold Spring Harb Perspect Med. 2014;4: a014241. 24890832 10.1101/cshperspect.a014241 PMC4031954

[7] Jorgensen I, Rayamajhi M, Miao EA. Programmed cell death as a defence against infection. Nat Rev Immunol. 2017;17:151–64. 28138137 10.1038/nri.2016.147 PMC5328506

[8] Mou Y, et al. Ferroptosis, a new form of cell death: opportunities and challenges in cancer. J Hematol Oncol. 2019;12:34. 30925886 10.1186/s13045-019-0720-y PMC6441206

[9] Zhao Y, Li Y, Zhang R, Wang F, Wang T, Jiao Y. The role of erastin in ferroptosis and its prospects in cancer therapy. Onco Targets Ther. 2020;13:5429–41. 32606760 10.2147/OTT.S254995 PMC7295539

[10] Liang C, Zhang X, Yang M, Dong X. Recent progress in ferroptosis inducers for cancer therapy. Adv Mater. 2019;31: e1904197. 31595562 10.1002/adma.201904197

[11] Koppula P, Zhuang L, Gan B. Cystine transporter SLC7A11/xCT in cancer: ferroptosis, nutrient dependency, and cancer therapy. Protein Cell. 2021;12:599–620. 33000412 10.1007/s13238-020-00789-5 PMC8310547

[12] Lin W, et al. SLC7A11/xCT in cancer: biological functions and therapeutic implications. Am J Cancer Res. 2020;10:3106–26. 33163260 PMC7642655

[13] Koppula P, Zhang Y, Zhuang L, Gan B. Amino acid transporter SLC7A11/xCT at the crossroads of regulating redox homeostasis and nutrient dependency of cancer. Cancer Commun (Lond). 2018;38:12. 29764521 10.1186/s40880-018-0288-x PMC5993148

[14] Slack FJ, Chinnaiyan AM. The role of non-coding RNAs in oncology. Cell. 2019;179:1033–55. 31730848 10.1016/j.cell.2019.10.017 PMC7347159

[15] Di Leva G, Garofalo M, Croce CM. MicroRNAs in cancer. Annu Rev Pathol. 2014;9:287–314. 24079833 10.1146/annurev-pathol-012513-104715 PMC4009396

[16] Zhou C, et al. Exosome-derived miR-142-5p remodels lymphatic vessels and induces IDO to promote immune privilege in the tumour microenvironment. Cell Death Differ. 2021;28:715–29. 32929219 10.1038/s41418-020-00618-6 PMC7862304

[17] Shrestha A, et al. MicroRNA-142 is a multifaceted regulator in organogenesis, homeostasis, and disease. Dev Dyn. 2017;246:285–90. 27884048 10.1002/dvdy.24477

[18] Stanley MA, Browne HM, Appleby M, Minson AC. Properties of a non-tumorigenic human cervical keratinocyte cell line. Int J Cancer. 1989;43:672–6. 2467886 10.1002/ijc.2910430422

[19] Li W, et al. HIVID: an efficient method to detect HBV integration using low coverage sequencing. Genomics. 2013;102:338–44. 23867110 10.1016/j.ygeno.2013.07.002

[20] Wang K, Li M, Hakonarson H. ANNOVAR: functional annotation of genetic variants from high-throughput sequencing data. Nucleic Acids Res. 2010;38: e164. 20601685 10.1093/nar/gkq603 PMC2938201

[21] Chen XJ, et al. A novel lymphatic pattern promotes metastasis of cervical cancer in a hypoxic tumour-associated macrophage-dependent manner. Angiogenesis. 2021;24:549–65. 33484377 10.1007/s10456-020-09766-2 PMC8292274

[22] Zhang H, et al. CAF secreted miR-522 suppresses ferroptosis and promotes acquired chemo-resistance in gastric cancer. Mol Cancer. 2020;19:43. 32106859 10.1186/s12943-020-01168-8 PMC7045485

[23] Eruslanov E, Kusmartsev S. Identification of ROS using oxidized DCFDA and flow-cytometry. Methods Mol Biol. 2010;594:57–72. 20072909 10.1007/978-1-60761-411-1_4

[24] Luo M, et al. miR-137 regulates ferroptosis by targeting glutamine transporter SLC1A5 in melanoma. Cell Death Differ. 2018;25:1457–72. 29348676 10.1038/s41418-017-0053-8 PMC6113319

[25] Wei WF, et al. Periostin^+^ cancer-associated fibroblasts promote lymph node metastasis by impairing the lymphatic endothelial barriers in cervical squamous cell carcinoma. Mol Oncol. 2021;15:210–27. 33124726 10.1002/1878-0261.12837 PMC7782076

[26] Miyake S, Murai S, Kakuta S, Uchiyama Y, Nakano H. Identification of the hallmarks of necroptosis and ferroptosis by transmission electron microscopy. Biochem Biophys Res Commun. 2020;527:839–44. 32430176 10.1016/j.bbrc.2020.04.127

[27] Deng YR, et al. Sp1 contributes to radioresistance of cervical cancer through targeting G2/M cell cycle checkpoint CDK1. Cancer Manag Res. 2019;11:5835–44. 31303791 10.2147/CMAR.S200907 PMC6610296

[28] Dixon SJ, et al. Ferroptosis: an iron-dependent form of nonapoptotic cell death. Cell. 2012;149:1060–72. 22632970 10.1016/j.cell.2012.03.042 PMC3367386

[29] Yang WS, Stockwell BR. Ferroptosis: death by lipid peroxidation. Trends Cell Biol. 2016;26:165–76. 26653790 10.1016/j.tcb.2015.10.014 PMC4764384

[30] Peng X, Zha L, Chen A, Wang Z. HOXA5 is a tumor suppressor gene that is decreased in gastric cancer. Oncol Rep. 2018;40:1317–29. 30015922 10.3892/or.2018.6537 PMC6072397

[31] Castro-Mondragon JA, et al. JASPAR 2022: the 9th release of the open-access database of transcription factor binding profiles. Nucleic Acids Res. gkab1113 (2021). 10.1093/nar/gkab1113 PMC8728201 34850907

[32] Shen Z, Song J, Yung BC, Zhou Z, Wu A, Chen X. Emerging strategies of cancer therapy based on ferroptosis. Adv Mater. 2018;30: e1704007. 29356212 10.1002/adma.201704007 PMC6377162

[33] Wang Y, Wei Z, Pan K, Li J, Chen Q. The function and mechanism of ferroptosis in cancer. Apoptosis. 2020;25:786–98. 32944829 10.1007/s10495-020-01638-w

[34] Li W, et al. Characteristic of HPV integration in the genome and transcriptome of cervical cancer tissues. Biomed Res Int. 2018;2018:6242173. 30018982 10.1155/2018/6242173 PMC6029443

[35] Thorland EC, et al. Human papillomavirus type 16 integrations in cervical tumors frequently occur in common fragile sites. Cancer Res. 2000;60:5916–21. 11085503

[36] Kim SH, et al. HPV integration begins in the tonsillar crypt and leads to the alteration of p16, EGFR and c-myc during tumor formation. Int J Cancer. 2007;120:1418–25. 17205528 10.1002/ijc.22464

[37] Ferber MJ, et al. Preferential integration of human papillomavirus type 18 near the c-myc locus in cervical carcinoma. Oncogene. 2003;22:7233–42. 14562053 10.1038/sj.onc.1207006

[38] Aldersley J, Lorenz DR, Mouw KW, D’Andrea AD, Gabuzda D. Genomic landscape of primary and recurrent anal squamous cell carcinomas in relation to HPV integration, copy number variation, and DNA damage response genes. Mol Cancer Res. 2021;19:1308–21. 33883185 10.1158/1541-7786.MCR-20-0884 PMC8349846

[39] Lin CY, et al. Transcriptional amplification in tumor cells with elevated c-Myc. Cell. 2012;151:56–67. 23021215 10.1016/j.cell.2012.08.026 PMC3462372

[40] Jiang C, Ito M, Piening V, Bruck K, Roeder RG, Xiao H. TIP30 interacts with an estrogen receptor alpha-interacting coactivator CIA and regulates c-myc transcription. J Biol Chem. 2004;279:27781–9. 15073177 10.1074/jbc.M401809200

[41] Zhang Y, Chen L, Yang S, Fang D. E2F1: a potential negative regulator of hTERT transcription in normal cells upon activation of oncogenic c-Myc. Med Sci Monit. 2012;18:RA12-15. 22207128 10.12659/MSM.882192 PMC3560676

[42] Li J, et al. KPNA2 promotes metabolic reprogramming in glioblastomas by regulation of c-myc. J Exp Clin Cancer Res. 2018;37:194. 30115078 10.1186/s13046-018-0861-9 PMC6097452

[43] Baluapuri A, Wolf E, Eilers M. Target gene-independent functions of MYC oncoproteins. Nat Rev Mol Cell Biol. 2020;21:255–67. 32071436 10.1038/s41580-020-0215-2 PMC7611238

[44] Garzon R, Fabbri M, Cimmino A, Calin GA, Croce CM. MicroRNA expression and function in cancer. Trends Mol Med. 2006;12:580–7. 17071139 10.1016/j.molmed.2006.10.006

[45] Li X, et al. miR-142-5p enhances cisplatin-induced apoptosis in ovarian cancer cells by targeting multiple anti-apoptotic genes. Biochem Pharmacol. 2019;161:98–112. 30639456 10.1016/j.bcp.2019.01.009

[46] Chen J, Jiang C, Du J, Xie CL. MiR-142-5p protects against 6-OHDA-induced SH-SY5Y cell injury by downregulating BECN1 and autophagy. Dose Response. 2020;18:710611448. 10.1177/1559325820907016 PMC7036514 32127787

[47] Lei P, Bai T, Sun Y. Mechanisms of ferroptosis and relations with regulated cell death: a review. Front Physiol. 2019;10:139. 30863316 10.3389/fphys.2019.00139 PMC6399426

[48] Ordóñez-Morán P, Dafflon C, Imajo M, Nishida E, Huelsken J. HOXA5 counteracts stem cell traits by inhibiting Wnt signaling in colorectal cancer. Cancer Cell. 2015;28:815–29. 26678341 10.1016/j.ccell.2015.11.001

[49] Ma HM, Cui N, Zheng PS. HOXA5 inhibits the proliferation and neoplasia of cervical cancer cells via downregulating the activity of the Wnt/β-catenin pathway and transactivating TP53. Cell Death Dis. 2020;11:420. 32499530 10.1038/s41419-020-2629-3 PMC7272418

[50] Yan Y, et al. SLC7A11 expression level dictates differential responses to oxidative stress in cancer cells. Nat Commun. 2023;14:3673. 37339981 10.1038/s41467-023-39401-9 PMC10281978

[51] Hu Y, et al. Crosstalk of ferroptosis and oxidative stress in infectious diseases. Front Mol Biosci. 2023;10:1315935. 38131014 10.3389/fmolb.2023.1315935 PMC10733455

[52] Park MW, et al. NOX4 promotes ferroptosis of astrocytes by oxidative stress-induced lipid peroxidation via the impairment of mitochondrial metabolism in Alzheimer’s diseases. Redox Biol. 2021;41: 101947. 33774476 10.1016/j.redox.2021.101947 PMC8027773

[53] Habib E, Linher-Melville K, Lin HX, Singh G. Expression of xCT and activity of system xc(-) are regulated by NRF2 in human breast cancer cells in response to oxidative stress. Redox Biol. 2015;5:33–42. 25827424 10.1016/j.redox.2015.03.003 PMC4392061

[54] Qin Z, et al. Upregulation of xCT by KSHV-encoded microRNAs facilitates KSHV dissemination and persistence in an environment of oxidative stress. PLoS Pathog. 2010;6: e1000742. 20126446 10.1371/journal.ppat.1000742 PMC2813276

