# Supplementary material for: HPV16 integration regulates ferroptosis resistance via the c-Myc/miR-142-5p/HOXA5/SLC7A11 axis during cervical carcinogenesis
Source: Cell Biosci. 2024 Oct 17;14:129. doi: 10.1186/s13578-024-01309-2 (PMC11484211; doi:10.1186/s13578-024-01309-2)
Supplement: Supplementary file 1 — Additional file 1 [file 13578_2024_1309_MOESM1_ESM.docx]

**Supplemental Figures**

**
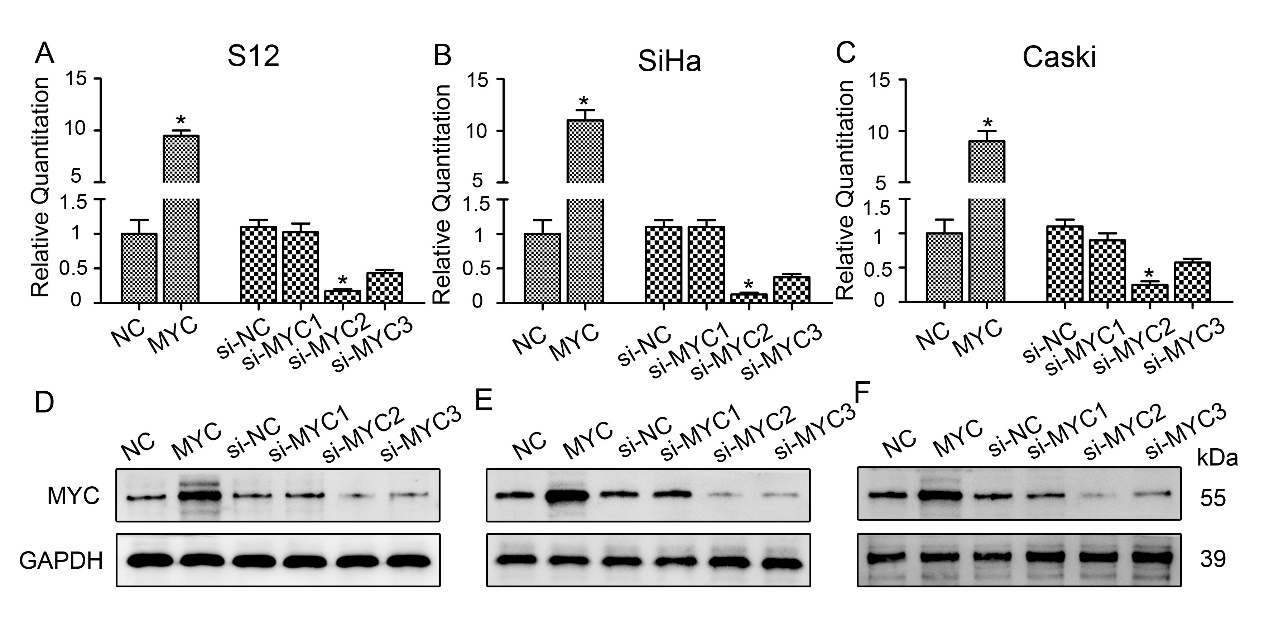
**

**Supplemental Figure 1.** RT-qPCR and western blot analysis demonstrating the level of c-Myc overexpression and knockdown in S12, SiHa, and CaSki cells. The most effective RNA interference effect was achieved with si-MYC2, which was subsequently utilized in the following experiments. **P*< 0.05.

**
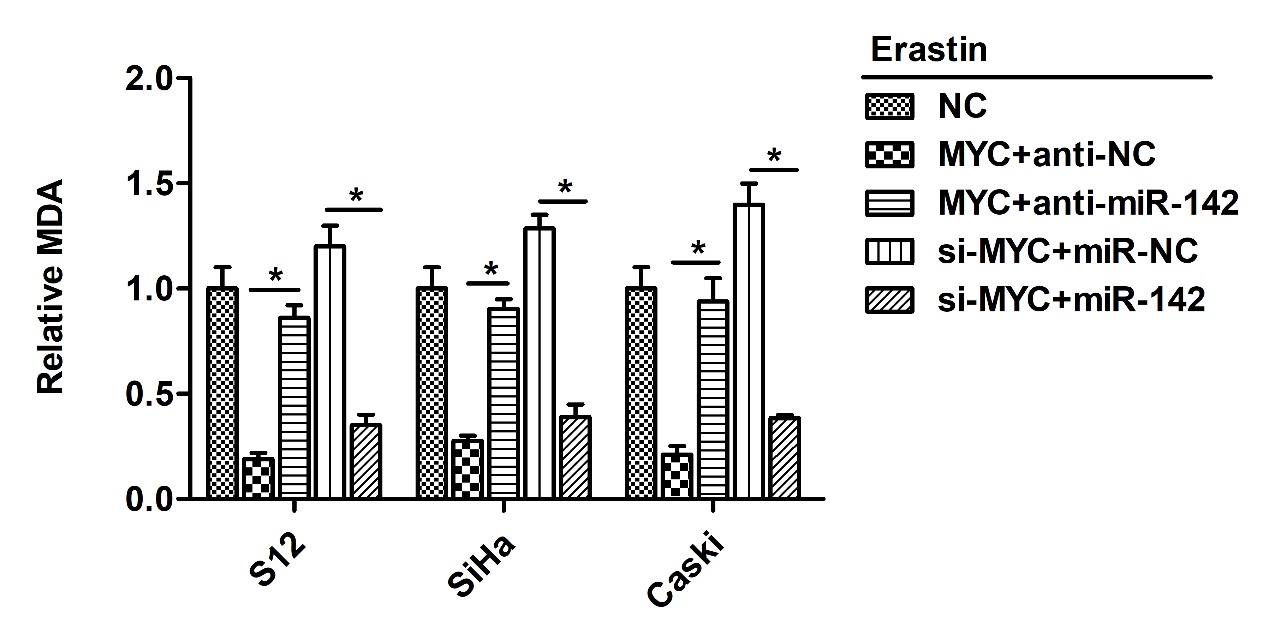
**

**Supplemental Figure 2.** HPV16-integrated cells (S12, SiHa and CaSki) were transfected with c-Myc overexpression vector with or without the miR-142-5p inhibitor, or with c-Myc siRNA with or without the miR-142-5p mimic. Subsequently, the cells were treated with 10 μM erastin for 24 hours, and the levels of MDA were quantified using a corresponding commercial kit. **P*< 0.05.

**
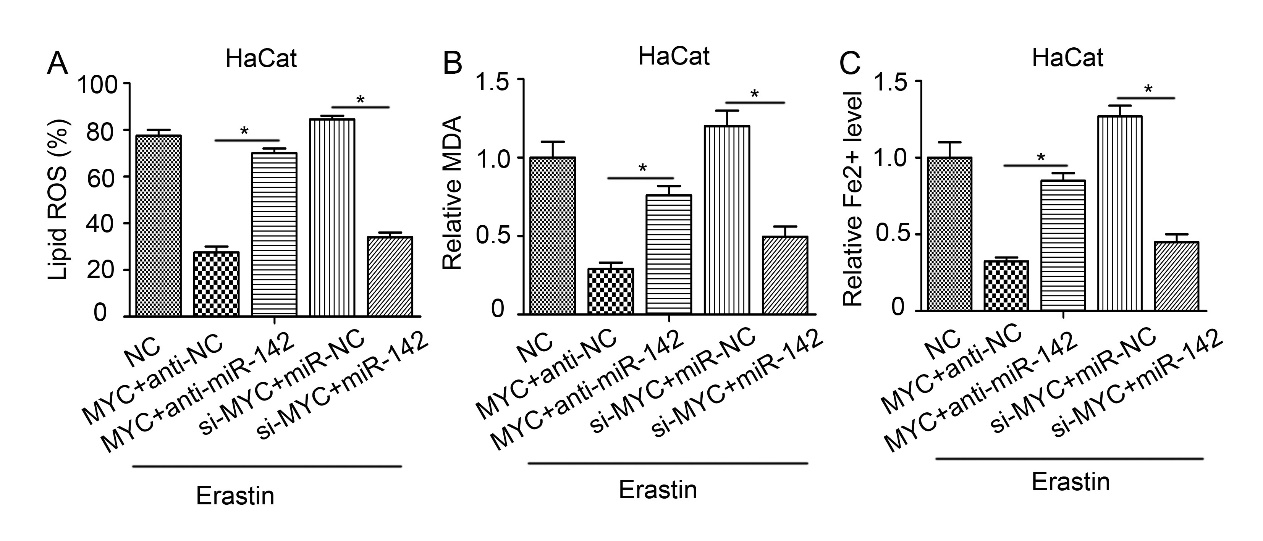
**

**Supplemental Figure 3.** HPV16 non-integrated HaCat cells were transfected with c-Myc overexpression vector with or without the miR-142-5p inhibitor, or with c-Myc siRNA with or without the miR-142-5p mimic. Subsequently, the cells were exposed to 10 μM erastin for 24 hours. Lipid ROS (A), MDA (B), and intracellular Fe2+ (C) levels were quantified using specific commercial kits. **P*< 0.05.

**
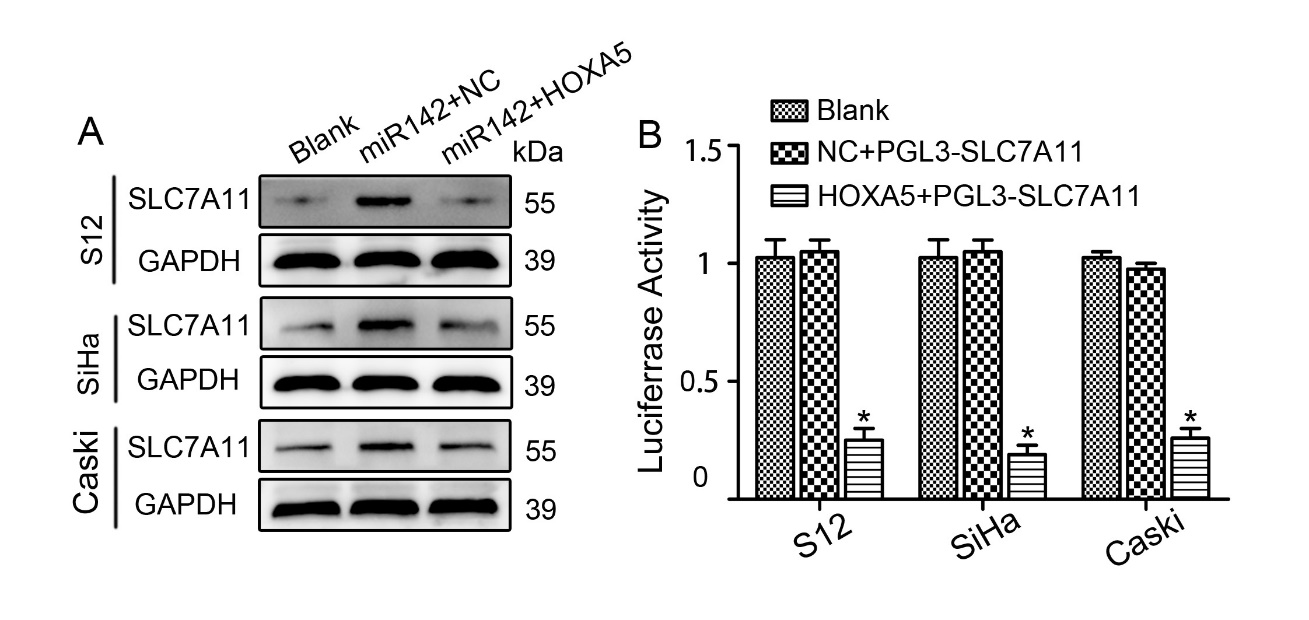
**

**Supplemental Figure 4.** (A) Western blot analysis demonstrating the expression of SLC7A11 in S12, SiHa and CaSki cells transfected with the miR-142-5p mimic with or without HOXA5 overexpression vector. **P*< 0.05. (B) Dual-luciferase reporter assays validated the direct interaction of HOXA5 with the SLC7A11 promoter region. **P*< 0.05.

**
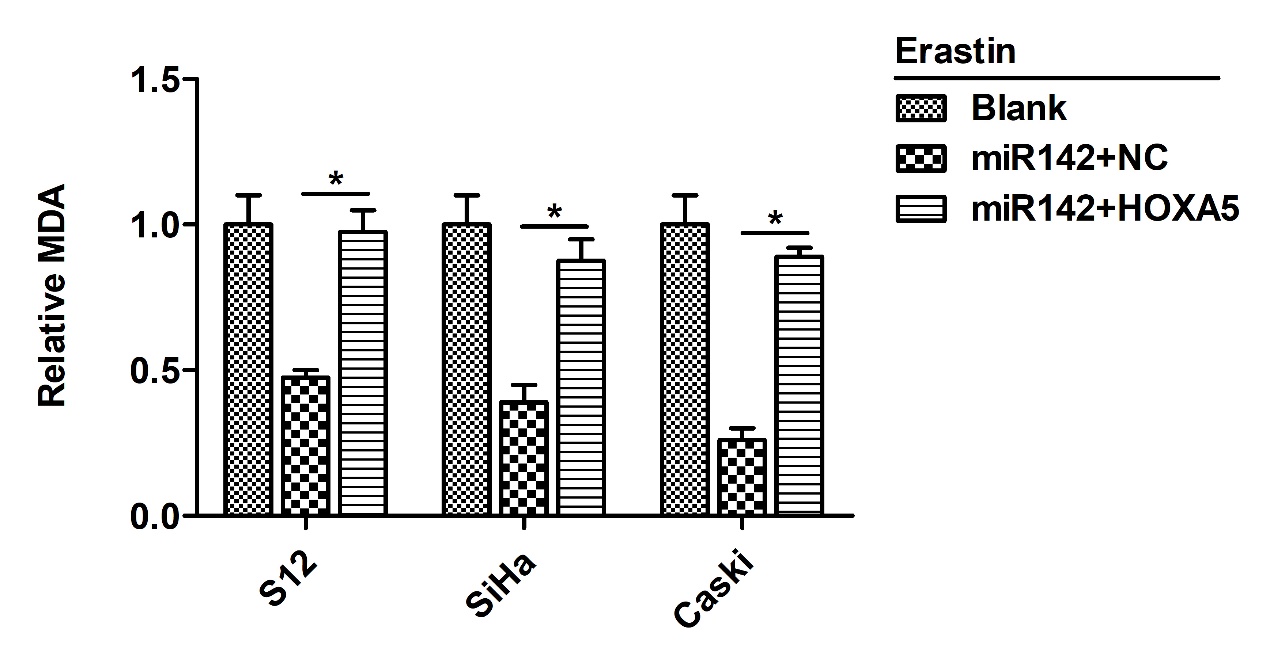
**

**Supplemental Figure 5.** HPV16-integrated cells (S12, SiHa and CaSki) were transfected with miR-142-5p mimic with or without HOXA5 overexpression vector, followed by treatment with 10 μM erastin for 24 hours. The levels of MDA were quantified using the appropriate commercial kit. **P*< 0.05.

**
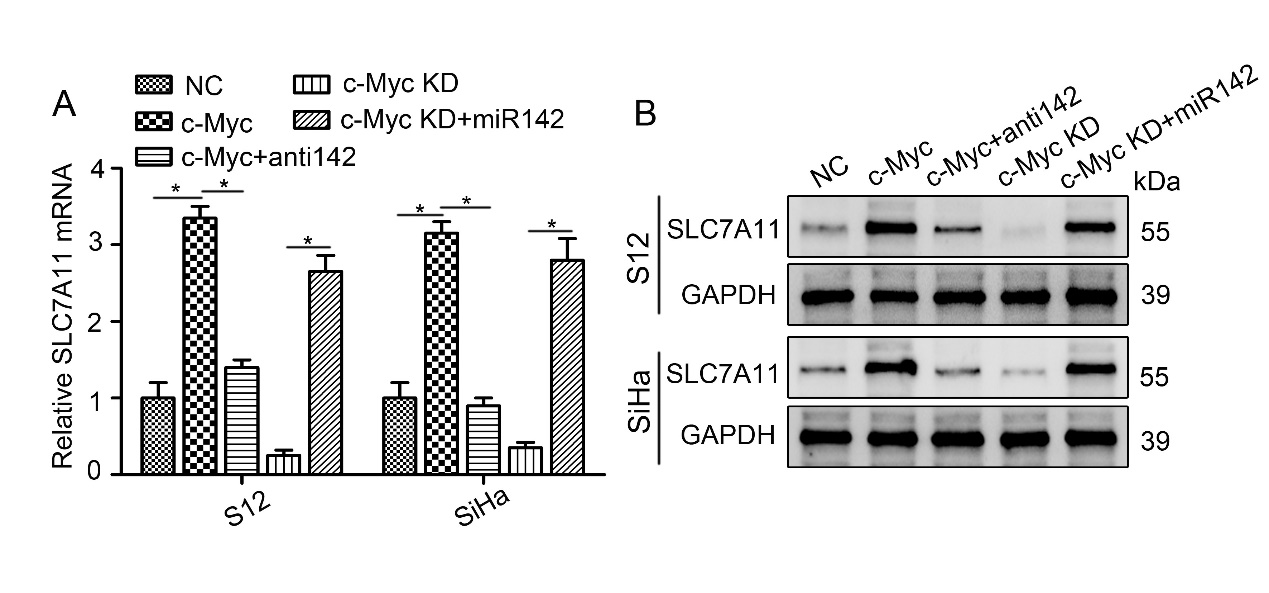
**

**Supplemental Figure 6.** RT-qPCR (A) and Western blot (B) results showing the relative expression level of SLC7A11 in the indicated S12 and SiHa cells described in Figure 5A. **P*< 0.05.

**Supplementary Tables**

**Table S1. Detailed primer sequences in the study**

|  | Forward | Reverse |
| --- | --- | --- |
| HOXA5 | AACTCATTTTGCGGTCGCTAT | TCCCTGAATTGCTCGCTCAC |
| SLC7A11 | GGTCCATTACCAGCTTTTGTACG | AATGTAGCGTCCAAATGCCAG |
| GAPDH | CCATCAATGACCCCTTCATTGACC | GAAGGCCATGCCAGTGAGCTTCC |
| HBS1 | CTATCTGAGTGGGGTCTTTGGCT | AGGACACTAGGAATCTCAGGGAACA |
| HBS2 | GAGTGGGGTCTTTGGCTCAAC | TGAGGTAAAAGTAGGACACTAGGAATCTCA |
| HBS3 | GTGGTGTCATTTTCTCAGGTGTCAAC | GCGAGAGGTTGGCAGGGAA |
| HBS4 | CAAAGGAACAACATGACAATGAAGGAT | TCACCATCCGAGTGTGACTAATACAAA |
| HBS5 | GTTGAGGAAGGCTTATAGTTGTGTGTATGT | CTGTTTTGTGAGTAGAATGTATCCTAAAGCTACA |
| HBS6 | TCTACTCACAAAACAGTCGCATGT | GCAACTCGTAGTGAGCAACAAAAG |

**Table S2**

**MIR142 (NR_029683-promoter):**

CTCTCCATCAAAACAAAACGAAACAAAACAAACTAGCAAAATAGGCTGTCCCCAGTGCAAGTGCAGGTGCCAGAACATTTCTCTATCGATAGGTACCATGCCCACATCCGGTGGCCGCCAGAACCTTCCCTCGAGCCTCTTTCCCGCCACTCCTCCAAGCCCCCTTCTCTCCGCATAGCACCTGTGCCCCACCTTAGGACCTGATCCTCACCCAGCTCTGCCCTGCCCTCTTGTCCCCACAGGTCCCAAAGAGAGCGTGAACACCTAGCCTAGTGCCATCAGTCCACCGGGCCTGAGAGTGTCCCCCGCACTGTGCCCTGCAGGCTGAGGCTGCAGGAGGGGTGGCTTTGTGGGGTGCTGAGGACAACAGTCTCCATGGACGCCCTTCATGCATCAGCACCTCCATGGCCCTCGGGAGCACACGCAGAGGAGCCAGGGCCTGGCCTGAGTGTTGGGAAGGGAAGGGAGTTTTCTGGGCTGAGGTCAGCAGCGGTGGGGCTCAGAGAGAAGGTCCTTTTCTTGTGCTGGAATTCTGGACTCCCCGTTTTGGGCACTCACCGAGGGCCCTGTCGAGGAGCCCTGGTCTTCTGGGGTCACCCTCAATGCCCTGAGTCAGTCCCAGGCTCTTGAGCCATCCCTTGGAGCTGGTGCCACGGGGGGAAGGGCAGCTGGGGCCGGAAGCCACCTGCCCAGGGCATGAGAGAGGGTCCCCAGCCCTCCCGCGGGCCCACCCCCTTCTGTTTCCTGTCAGTCTGTCCTCCCCAGGGAGGGAGGGAGGGAGGGCGCTCATCCCCTGCGGGCGGGGGCGGGGCGCGCTCTGAGACCGTTGGGCGCCCTGAGATCTGGCCCACGTGGCCTCGGGGTTATAAGCCCTGCCCTCCCTGAAGGGAACCCCACTTCGGAGCCTGGAGCACAGGGCGAGCTCTCCTCCGCCCTGCAGCCTCAGGTAAGGATCCGGACCTTCACTGCTGCCCCAACTGGGGCTCCTCAGCTCGGGGCCTCTAGGGGCTTGGGCTGCTGGTCTGGGTGGAGGCGCGTTGGGGGTGTCGAGGAGCTTGGGTGCAAGCCCCATGCGGGAAGCTCAGGGCAAAGCGCCTTGAAACCCTCTCTAGTGAAGGAAGGTAAAATGGCTCCGGGACCCCAGGCGGGGAAAGAGGTGTCCAAGGAGGAGGGCTGGAGAGTAAGGCGGGGATGGCAGAGGGTACGCAGAGGCTGAGAAGTGGGACCAGTGTCTCAAGGGGCGCCAGCCTCTTCCTCCGCCCAACATGGCCACCATTTTTGTTGGGATAGCCTTGGGCTCCTGCCAGGGGAACAGCAGGGCAGTGAGGGAGTCAGGACTTTGGGCTGGTGGGGAGGCCGAGAATCAAGGCTCTGGGGAGTCAGGACTTTGGGGCTGGTGGAGTGGCTGGGGACTGAGGCCCTGGGGGGTCAGGACCTTGGGCTGCTATGGAGGCTGGGACTGAGGCTCTGGGGGAGTCAGGACCTTGAGCTGGTGGGGAGGCTGGGAATTGAGGCCCTGGTGTGTTCAAGACCTTGGGCTGGTGGGGAGGCCAGGGCCTGAGGCCCTGGGGGGTCAGGACCCTGGGCTGTTGGGGAGGCTGGGGACTTAGGCCCTGGGGGGTCAGGATCTTGGGCTGGTGGGGAGGCTGAAGGGTGGGGACTGAGGCTCTGGGCAGTCAGGACCTCACGGTTGGGGTGGACTGGAGACCAAGACCTTGGCAGGGGAGCTGTGGCTGCCTCATTTGGACGCTGGAGGGTGGCTAGCGTGGCTGGAAGCGGCCAGCCAGGGGTTCACAGAACTGAAGGTGAGGCCTCCAGAGGCCCTAGTCTCTACCTGAGTGTCTCTGAAACTGGGGGGATGGGGTGGAGCCTTTAGGGGGAAGGGAAGAGGGAACTGAAGAGGAAGTGGGGGAGGGAGGTAGAGGAGGCAAGTCTGGCGCCATGCTGAGTCACCGCCCACAAGGCCCAGGGCGGGCCCTCGGGGGGCCCTGGCAGGGTTGGGGGGATCTTAGGAAGCCACAAGGAGGGCTGGGGGGCTCTTGGAGCAGGAGTCAGGAGGCCTGGGCAGCCTGAAGAGTACACGCCGACGGACAGACACTCGAGATCTGCGATCTAAGTAAGCTTGGCATTCCGGTACTGTTGGTAAAGCCACCATGGAAGACGCCAAAAACATAAAGAAAGGCCCGG

**HOXA5 (NM_019102-3utr(miR-142-5p)-wt):**

AATCAGAGAGATCCTCATAAAGGCCAAGAAGGGCGGAAAGATCGCCGTGTAATTCTAGATGTTGTAGAGGTGACTTGATGAGACACAACTTGTTCGACGTGTAGTGACTAGTGACTCTGTGATGAAAACTGTGACTCCAAGCGGTGTGTCCCTGCGTGCCTTTATAGGACCCTTTGCACGAACTCTGGAAGTGGCTCTTATAAGCGCAGCTTCAGTGATGTATGTTTTTGTGAACAAAGTTACAAATATTGTCCAAGTCTGGCTGTCTAGAGTCGGGGCGGCCGGCCGCTTCGAGCAGACATGATAAGATACATTGATGAGTTTGGACAAACCACAACTAGAATGCAGTGAAAAAAATGCTTTATTTGTGAAATTTGTGATGCTATTGCTTTATTTGTAACCATTATAAGCTGCAATAAACAAGTTAACAACAACAATTGCATTCATTT

**HOXA5 (NM_019102-3utr(miR-142-5p)-mut)：**

AGATCCTCATAAAGGCCAAGAAGGGCGGAAAGATCGCCGTGTAATTCTAGATGTTGTAGAGGTGACTTGATGAGACACAACTTGTTCGACGTGTAGTGACTAGTGACTCTGTGATGAAAACTGTGACTCCAAGCGGTGTGTCCCTGCGTGCAGGGCGAGGACCCTTTGCACGAACTCTGGAAGTGGCTCTTATAAGCGCAGCTTCAGTGATGTATGTTTTTGTGAACAAAGTTACAAATATTGTCCAAGTCTGGCTGTCTAGAGTCGGGGCGGCCGGCCGCTTCGAGCAGACATGATAAGATACATTGATGAGTTTGGACAAACCACAACTAGAATGCAGTGAAAAAAATGCTTTATTTGTGAAATTTGTGATGCTATTGCTTTATTTGTAACCATTATAAGCTGCAATAAACAAGTTAACAACAA

**SLC7A11(NM_014331-promoter):**

TTTCTCTATCGATAGGTACCATTCTGAGTGGTGGCCTCTATCTTACATGAAAGTACAATCCTTTTGGTTTTTAGTCTAACATCAGCCATTTTTCTTCATAATTAAAAAAAAAATACTGTGAGGAGGAAAATGTTATTTAATATTGAAAGAATAATCGTCCTTTATAATTCATTCAGTTTCCTATCTGAGTGGGGTCTTTGGCTCAACTTATGAGTTCAGTTATTTATAACAAAATTTAAAATTAAAGTGTGACACTGTGTTAATTAGAGAAAGTTGCTGTTCCCTGAGATTCCTAGTGTCCTACTTTTACCTCATATTTCTTCAGAGCTAATATGTTTCTCTTACTGGGAAAGAAGAGCTGCTCCTTAGGTCCATTAATCAGGAAAATTGTGTGAGTGAAAGGAGATGTAGGAGGAGGAAGAGAATTGGAAAGAAAAGAATCTCTATTTAGCTTCCTTCTTTGGGAGTTTTAGCAGGATTGCATGAGGAAAATGATCCATTTTTGTTTTTTGAAAGACATTAACAACTGAGAAAAAGTATTTATTTTGGTTTTATAAAAACTTTAACTTGGTGTAGCTGAAACCAATTATATAGAATTTAGATAGATAATTTAAAAAGGAGAAAATGTAGCCTCAATTATTTGGATGGCAAAGCATTGAGGTGGTGTCATTTTCTCAGGTGTCAACATAACAGAAAATGGTTCTGACCTTTTCCATGCTACTTTCCTAATGGCGATTTCTCATTTCTTTCTTTGATCATCCTCACCATACTTGACTCCCTTCTTTCCCTGCCAACCTCTCGCACACAGGCTTTTTGTTTGTGGTTTTTGTTCAAAGGTTCAGAAGCTTATTTAATGGTGCGTATAAAGGTAGCTTCAGGGTAATGATAACATTCAATTCTTCTCACTGAATCCAAAACCACATGTAACTATAGCCAAAAATGATATCTAGGATTTCTTTTCTTTTCATATTTGTTTTTTCTTGACACTTGGTTGAGAAAGTTAGAAGGGTTTAAAAATGTTTTGCCTTTTTGACTATTGCAAAGTAAATATTTTAATTTAGCTTACATTTTTAATGCATTTTAAATTTATAATAATAACAATAGTTTATATAATGTGGAAAAAAGTCATACATAATATCATTACTTTCTATAACTACCATTCTGTTTTCCTTTACAGGCTTTTGCTATATACATATTTTTATGTAATTGTAATTGTGTAATTTTGAACTTAGAAATTTTTTTTGGTAATTCAAAGGAACAACATGACAATGAAGGATATGTATCTTTTACATAGTTCAAAAAAATCACCTAGTGCTAATGAGAATCAGAAAATGACTATTTTCTGGAGTCATGGTGAATTTTGTATTAGTCACACTCGGATGGTGATTTAAAATACTGGTTTATTATGAGTAGTAAGAAATAATTTTATCTTTTAATGTTGAGGAAGGCTTATAGTTGTGTGTATGTGACAGAAGTATAAAGTGTAGAATCTTTAATTTTGTAAATATTGGATTTGACTGTATTGCCTTATAAAAAACTCAAAATAAAAAAATAAAAAGCATTACACAAATTATGAAATTCAAACTGTGTTGTTATAAACAATGTAGCTTTAGGATACATTCTACTCACAAAACAGTCGCATGTAAAATGTTTTTATACACGCATTAAAGAAAAAGAAACTCCTTAAAATGAAGTAACTATTTCCTGTTTCATTTTGTTTGAACAGCTTTTGTTGCTCACTACGAGTTGCTTTAAATCTCTGGGAAGGTCTGTTCCGAATTTACTACTTCTGGATTGGCTAAAATCTCTTTAAAGTGTGTGCTTTGTTCTCTAAAAAGCTTAGGTCAGTTGAGCAACAAGCTCCTCCTGTTTTTTTCTTTTTTTAAAAAAAAGAGCTGAGTAATGCTGGAGGCTTCTCATGTGGCTGATGCAAACCTGGAGAATTTGCATCATCATTTAGCTGTAGTAAGTTGGTGTGACAGGCAGGCGCTTAAATACAAGCCCATGAGGAAGCTGAGCTCTCGAGATCTGCGATCTAAGTAAGCTTGGCATTCCGGTACTGTTGGTAAA
